# Inhibition of the RhoGTPase Cdc42 by ML141 enhances hepatocyte differentiation from human adipose-derived mesenchymal stem cells via the Wnt5a/PI3K/miR-122 pathway: impact of the age of the donor

**DOI:** 10.1186/s13287-018-0910-5

**Published:** 2018-06-19

**Authors:** Diana Chaker, Charbel Mouawad, Albert Azar, Didier Quilliot, Ibrahim Achkar, Ziad Fajloun, Nehman Makdissy

**Affiliations:** 1 Lebanese University, Doctoral School for Sciences and Technology, Laboratory of Applied Biotechnology, Azm Center for Research in Biotechnology and its Applications, Tripoli, Lebanon; 2 Reviva Regenerative Medicine Center, Human Genetic Center, Middle East Institute of Health Hospital, Bsalim, Lebanon; 3 Paris Saclay University, Doctoral School, Therapeutical Innovation, Inserm UMR935, Villejuif, France; 4 Independent researcher, Kfarzeina, Zgharta, Lebanon; 5 Diabetologia-Endocrinology & Nutrition, CHRU Nancy, INSERM 954, University Henri Poincaré de Lorraine, Faculty of Medicine, Nancy, France; 6 Achkar Clinics, St. Elie Center, Antelias, Lebanon; 7 Lebanese University, Faculty of Sciences III, Department of Biology, Kobbe, Lebanon

**Keywords:** Aging, Cdc42, ML141, Adipose derived mesenchymal stem cells, Hepatocyte differentiation, Exosomes release, miR122, Wnt, MAPK, PI3K

## Abstract

**Background:**

Human adipose-derived mesenchymal stem cells (hADSCs) are promising cells that may promote hepatocyte differentiation (Hep-Dif) and improve liver function, but the involvement of Cdc42, a key small RhoGTPase which plays a crucial role in aging, is still not well established. We hypothesized that the inhibition of Cdc42 may rescue the hepatogenic potential of hADSCs derived from aged donors.

**Methods:**

hADSCs isolated from 61 women of different ages were cultured for evaluation of the proliferation of cells, adherence, apoptosis, immunomodulation, immunophenotyping, multipotency, gene expression, and cell function during Hep-Dif. Inhibition of Cdc42 by ML141 was realized during two phases: initiation (days –2 to 14 (D–2/14)) from undifferentiated to hepatoblast-like cells, or maturation (days 14 to 28 (D14/28)) from undifferentiated to hepatocyte-like cells. Mechanistic insights of the Wnt(s)/MAPK/PI3K/miR-122 pathways were studied.

**Results:**

Cdc42 activity in undifferentiated hADSCs showed an age-dependent significant increase in Cdc42-GTP correlated to a decrease in Cdc42GAP; the low potentials of cell proliferation, doubling, adherence, and immunomodulatory ability (proinflammatory over anti-inflammatory) contrary to the apoptotic index of the aged group were significantly reversed by ML141. Aged donor cells showed a decreased potential for Hep-Dif which was rescued by ML141 treatment, giving rise to mature and functional hepatocyte-like cells as assessed by hepatic gene expression, cytochrome activity, urea and albumin production, low-density lipoprotein (LDL) uptake, and glycogen storage. ML141-induced Hep-Dif showed an improvement in mesenchymal-epithelial transition, a switch from Wtn-3a/β-catenin to Wnt5a signaling, involvement of PI3K/PKB but not the MAPK (ERK/JNK/p38) pathway, induction of miR-122 expression, reinforcing the exosomes release and the production of albumin, and epigenetic changes. Inhibition of PI3K and miR-122 abolished completely the effects of ML141 indicating that inhibition of Cdc42 promotes the Hep-Dif through a Wnt5a/PI3K/miR-122/HNF4α/albumin/E-cadherin-positive action. The ML141(D–2/14) protocol had more pronounced effects when compared with ML141(D14/28); inhibition of DNA methylation in combination with ML141(D–2/14) showed more efficacy in rescuing the Hep-Dif of aged hADSCs. In addition to Hep-Dif, the multipotency of aged hADSC-treated ML141 was observed by rescuing the adipocyte and neural differentiation by inducing PPARγ/FABP4 and NeuN/O4 but inhibiting Pref-1 and GFAP, respectively.

**Conclusion:**

ML141 has the potential to reverse the age-related aberrations in aged stem cells and promotes their hepatogenic differentiation. Selective inhibition of Cdc42 could be a potential target of drug therapy for aging and may give new insights on the improvement of Hep-Dif.

**Electronic supplementary material:**

The online version of this article (10.1186/s13287-018-0910-5) contains supplementary material, which is available to authorized users.

## Background

Aging is a process that results from an increased failure in a system normally designed for growth and reproduction [1], and is a major risk factor for most chronic diseases. Among organs having remarkable abilities for regeneration is the liver, where aging is correlated with changes to the normal liver and in liver diseases and is associated with continuing alteration of hepatic functions [2]. Stem cell aging could lead to the collapse of tissue function and regeneration in older humans [3]. Although specialized niches usually protect stem cells from age-dependent stress mechanisms, these cells can seemingly grow old [4]. Current research is focusing on changing any feature that could possibly slow down the progression of aging, and mesenchymal stem cells (MSCs) [5] have emerged as a promising tool and an attractive stem cell source for this purpose. While the differentiation potential of MSCs is comparatively limited to that of embryonic stem cells or induced pluripotent stem cells, they are a much safer source regarding the risk of inducing teratoma and raise less ethical debates when it comes to clinical applications, particularly in liver diseases [6]. Recent strategies in tissue engineering and cell therapy have shown the efficacies of human adipose-derived MSCs (hADSCs) in regenerative medicine [7] and that hADSCs hold numerous benefits over bone marrow-derived MSCs (BMSCs) with higher potentials for proliferation and differentiation capacities in vitro [8]. Indeed, once induced into functional hepatocyte-like cells (HLCs), hADSCs provided a promising nontransgenic tool for autologous, hepatocyte-based therapies by displaying the in-vitro functions of mature hepatocytes and showing efficient repopulation properties in mice models of liver injury [7, 9]. Nevertheless, the age of the hADSCs donors presents a negative impact on the properties of ADSC expansion, differentiation, doubling time, homing, and immune modulation [10–13]. Age-dependent MSC senescence has been linked to a decrease in mitochondrial activity, high expression of p16^INK4a^, p53, and p21, and an increase of β-galactosidase and reactive oxygen species (ROS) accumulation [5, 14–16].

Recent findings have shown that the functional decline in the elderly hematopoietic stem cells (HSCs) was associated with an upregulation of the activity of the cell division cycle 42 (Cdc42) protein, a small G-protein that belongs to the RhoGTPase family of the Ras superfamily and regulates actin cytoskeleton, cell polarization and adhesion, migration of stem cells, and tissue regeneration [17, 18]. Mice HSCs showed elevated Cdc42-GTP expression after genetic deletion of a negative regulator of Cdc42 and exhibited a severe early aging pattern [19, 20]. Several reports discussed the role of Cdc42 in human MSC proliferation, migration, and differentiation [21, 22]. Therefore, the need to inhibit Cdc42 becomes interesting. The histone deacetylase inhibitor Trichostatin A (TSA) was shown to be a nonspecific inhibitor of Cdc42 activity but a key factor for MSC differentiation into hepatocytes via the induction of microRNA-122 (miR-122) [23, 24]. Other studies indicated that the pharmacological and irreversible inhibition of Cdc42 activity by CASIN (a Cdc42 activity-specific inhibitor) was able to overturn aging and restore the spatial distribution of histone H4 lysine 16 acetylation of aged HSCs to a status similar to that seen in young HSCs [25, 26]. Thereby, upon transplantation, CASIN-treated HSCs were shown to be potentially identical to HSCs isolated from young donors [19]. More recently, ML141 was shown to be a novel, potent, and noncompetitive allosteric inhibitor of Cdc42, promoting migration and regulation of mice HSC polarity with a notable low potency of inhibition against other members of the Rho family [27, 28]. Currently, there are no published data showing the impact of Cdc42 inhibition on the rejuvenation of hADSCs from old donors, and particularly the impact of ML141.

In addition to Wnt(s) signaling, Cdc42 may crosstalk with several pathways, including the phosphatidylinositol-3 kinase (PI3K)-protein kinase B (PKB) pathway, Janus kinase (JAK)-signal transducer and activator of transcription (STAT) pathway, and extracellular signal-related kinase (ERK), JNK, and p38 mitogen activated protein (MAP) kinases, where the PI3K/PKB, MAPK, and WNT signaling pathways were shown to be involved in hepatocyte differentiation (Hep-Dif) [29–31]. In fact, several reports have reviewed the key features of stem cell aging biology and described several pathways implicated in the process of stem cell aging: Wnt signaling, oxidative stress, ROS, apoptosis, p53, PI3K, MAPK, nuclear factor kappa beta (NF-kB), microRNA-related pathways, etc. [32–34]. Furthermore, PI3K can be triggered by insulin receptor substrate (IRS) ligand binding to insulin-like growth factor (IGF), thus activating the JNK pathway and subsequently Cdc42-GTP binding; it has also been reported that insulin and IGFs enhance Hep-Dif from human embryonic stem cells via the PI3K/PKB pathway [30]. On the other hand, Wnt(s) signaling had been associated with Hep-Dif through a Wnt/β-catenin pathway inhibition, thus promoting Hep-Dif [35–38]. Repression of Wnt/β-catenin signaling in the anterior endoderm is essential for liver development and the induction of the hepatobiliary differentiation toward hepatocytes [37]. Inducing the translocation of β-catenin to the nucleus increased cell proliferation, and its stabilization alone leads to increased propensity toward cholangiocytes over hepatocytes [39]; otherwise, the Wnt pathway is the major regulator of polarity and cell fate specifications [40–42].

Recent studies have uncovered profound roles for a family of microRNAs (miRNAs) controlling gene expression in almost every biological process including development, aging, and cell death, but also in the control of diverse aspects of hepatic function and dysfunction, and these have emphasized the role of the most abundant miRNA in human liver, miR-122, a key factor and therapeutic target in liver disease [43–45]. Loss of miR-122 has been associated with hepatocellular carcinoma [46] and miR-122 has been considered to be an essential host factor for hepatitis C virus replication [47]. Concerning its role in Hep-Dif, miR-122 was reported to be a direct target of the liver-enriched transcription factor (LETFs) hepatocyte nuclear factor (HNF)4, which controls Hep-Dif [48], and its overexpression promotes Hep-Dif through a miR-122/HNF4α-positive feedback loop [49, 50]. Among the signaling pathways that control miR-122, PI3K/PKB has been demonstrated to positively regulate miR-122 [51].

In this study, we describe that ML141 succeeded in decreasing the Cdc42-GTP levels in hADSCs derived from aged donors and promoted their hepatocyte-like cell generation in a manner that is functionally equivalent to hADSCs derived from younger subjects, and we further elucidate the involved mechanism.

## Methods

### Population design

**Fig. 1:**
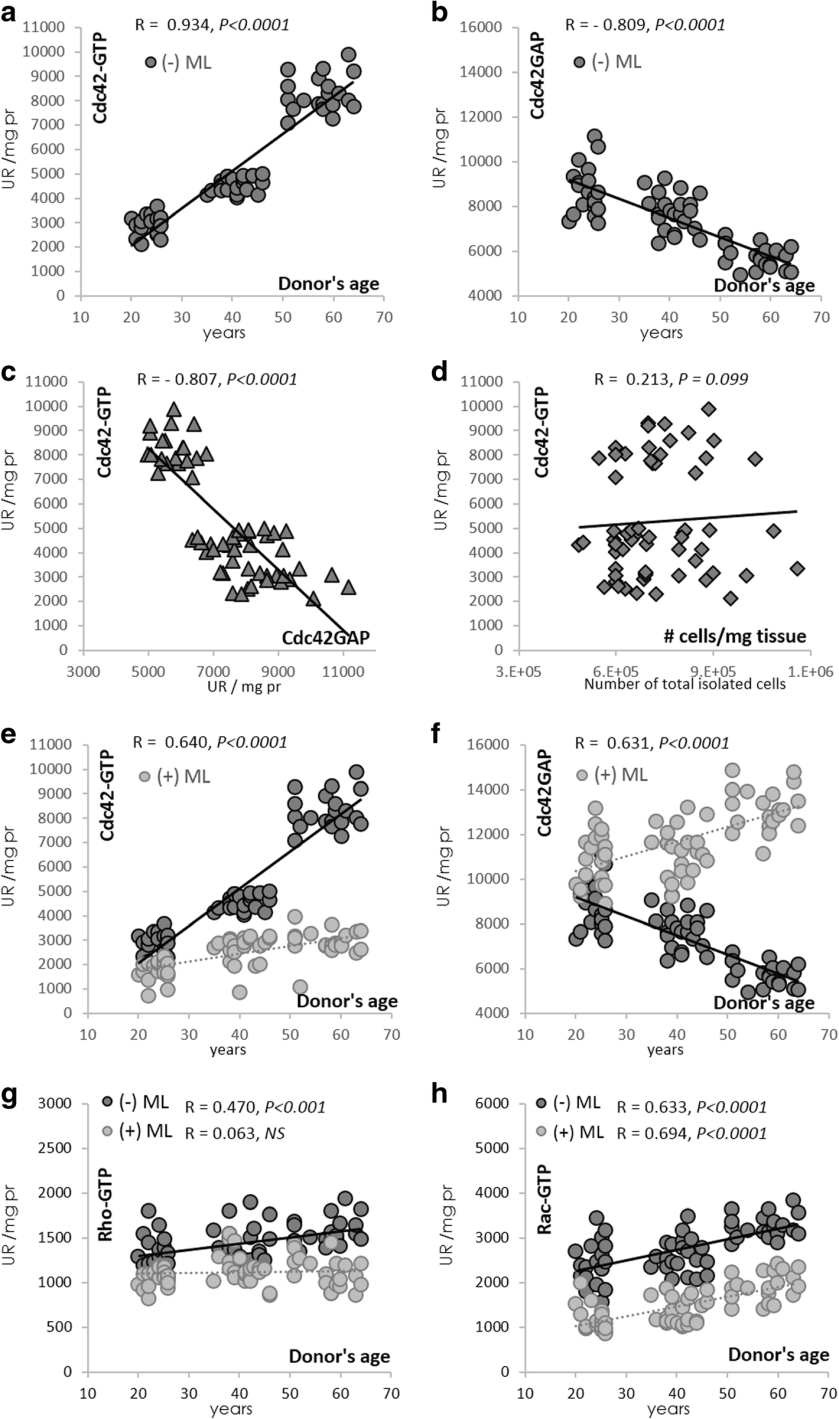
Cdc42 activity increases with age. hADSCs were isolated from lipoaspirates and cultured for 96 h and then treated for 24 h with or without the Cdc42/Rac1 GTPase inhibitor ML141 (ML; 10 μM). Cell lysates from undifferentiated hADSCs were used for the Pull-down assays of Cdc42-GTP (500 μg) and Cdc42GAP (100 μg). Correlations between donor age and Cdc42-GTP (**a,e**), donor age and Cdc42GAP (**b,f**), Cdc42-GTP and Cdc42GAP (**c**), Cdc42-GTP and cell yield/mg of collected adipose tissue (**d**), donor age and Rho-GTP (**g**), and donor age and Rac-GTP (**h**). Results represent the mean ± SEM of two independent experiments realized on the 61 subjects after normalization to GAPDH. Pearson correlation coefficient (*R*) and the *P* value are shown

Sixty-one healthy subjects were enrolled in this study and were classified into three age groups: young (23.8 ± 0.4 years), middle-aged (40.8 ± 0.6 years), and old/aged (57.6 ± 0.9 years). This classification was based on the distribution of the population as reported in Fig. 1a. Adipose tissue harvests (< 600 mg) collected from the abdominal area were processed manually for stromal vascular fraction (SVF) isolation. The cell yield (total nucleated cells and MSC numbers) showed no significant differences between the different age groups (Additional file 1: Table S1). Subjects were included in the study only if they had no notable pathological history, in particular liver diseases, and were excluded based on a list of criteria as shown in Additional file 1: Table S1.

### Isolation and expansion of hADSCs

Samples of human adipose tissue (200 ml or ~ 100–300 mg) were obtained by lipoaspiration or biopsy from abdominal subcutaneous fat, and then processed for the isolation of SVF and culture of ADSCs as described previously [52]. The hMSCs (hADSCs) were isolated by their ability to adhere to the culture flask. The first medium change removed the nonadherent cells after 3 days of culture. Cells were used in passage 3 to avoid the risk of transdifferentiation and spontaneous transformation. The hepatocyte/adipocyte/neural differentiation was induced at the third passage where all the cells had > 98% mesenchymal phenotype of a homogenous population of hADSCs and after confirming the absence of any chromosomal abnormality as determined by karyotyping.

### Hepatogenic, adipogenic, and neurogenic induction of hADSCs

hADSCs (10^6^ cells) were seeded into MaxGel™ extracellular matrix (ECM)-coated plates and triggered for differentiation at day 2 postconfluence (designated as day 0) for a period of 28 days. Four groups were studied: young, aged, and aged treated with ML141 (5 μM) from day −2 to 14 (D−2/14), or 14 to 28 (D14/28). Different cocktails of inducers were supplemented to the culture media depending on the studied lineage. Medium without inducers served as the negative control experiments. Media were changed every 3 days. All growth factors, hormones, and supplements were purchased from Sigma Aldrich. Cell morphology and cytotoxicity were controlled daily. Cell differentiation to the multilineage was microscopically supervised and controlled for each lineage as defined below.

#### Hepatocyte differentiation (Hep-Dif)

All groups underwent the same Hep-Dif protocol: 1) preinduction at confluence (day −2) where hADSCs were cultured in serum-free medium for 48 h with 20 ng/ml basic fibroblast growth factor (b-FGF) and 20 ng/ml epidermal growth factor (EGF); 2) induction from day 0 to 14 of the differentiation using media free of serum and supplemented with 30 ng/ml hepatocyte growth factor (HGF), 1× iTS and 10^−8^ M dexamethasone; and 3) maturation from day 14 to 28 of the differentiation using media free of serum supplemented with 50 ng/ml OSM, 20 ng/ml HGF, 1× iTS, and 1 μM dexamethasone. Cells exhibited hepatoblast-like cell (HBLC) and hepatocyte-like cell (HLC) phenotypes at D14 and D28, respectively. Hepatocyte lineage was confirmed by the expression of hepatic markers cytokeratin (CK)-18, albumin (ALB), and alpha-fetoprotein (AFP).

#### Adipocyte differentiation (Adp-Dif)

hADSCs (10^6^ cells) were induced with the Adp-Dif medium (control medium supplemented with insulin (10 μM), dexamethasone (1 μM), indomethacin (200 μM), and 3-isobutyl-1-methylxanthine (500 μM)) at day 0 of the differentiation. Cells exhibited immature adipocyte-like cell (IALC) and mature adipocyte-like cell (MALC) phenotypes at D14 and D28, respectively. Adipocyte lineage was confirmed by the presence of lipid droplets as identified by Oil Red O staining and the expression of adipogenic markers PPARγ and Pref-1 (key early positive and negative regulator of adipogenesis, respectively), and FABP4 (responsible for the formation of mature adipocytes) [53].

#### Neural differentiation (Neu-Dif)

This procedure consisted of two steps: 1) MSCs were cultured in a neurobasal (NB) medium (Hyclone advanced basal medium stem) supplemented with 10% serum (Hyclone advanced stem cell growth supplement), 2% B27, 1% PSA at 37 °C, and 5% CO_2_ (days 0–14); 2) NB medium was supplemented with 20 ng/ml b-FGF, 20 ng/ml EGF, and 20 ng/ml β-NGF for 14 days (days 14–28). Neural lineage was observed first at D14 by the induction of hMSCs to differentiate into neurosphere (NSP)-like structures and second at D28 by a final differentiation into neuron-like cells (NLCs), and controlled by the expression of neurogenic markers (NeuN for neurons, O4 for oligodendrocytes, and GFAP for astrocytes) [54].

### Quantitative real-time polymerase chain reaction (RT-qPCR)

RNA was extracted from the cell pellets using the RNAspin Mini kit (GE Healthcare) according to the manufacturer’s instructions. The mRNA was reverse transcribed to complementary DNA (cDNA). cDNA was amplified using VeriQuest Fast SYBR Green qPCR Master Mix (75,690 500 RXN, Affymetrix). Thermal cycling was performed on a LightCycler 2.0 (Roche) with the following protocol: 1 cycle (50 °C/2 min), 1 cycle (95 °C/5 min), 45 cycles (95°C/3 s and 60°C/30s). The analysis of the melting curve was performed to exclude nonspecific amplification products. Relative changes in expression were calculated after normalization to GAPDH. Primers used are listed in Additional file 1: Table S1.

### Cdc42 siRNA transfection

MSCs at 70% of confluence were cultured (10^5^ cells) and transfected with Lipofectamine RNAiMAX (Thermofisher Scientific) according to the manufacturer’s instructions. MSCs were transfected with small interfering (si)RNA-Lipofectamine RNAiMAX complexes (respectively, 6000 pmol and 40 μl in 2 ml medium) at day –2 (D–2) and the cells were induced to differentiate with the neuronal induction medium at day 0 (D0). To maintain the silencing effect, an additional dose of siRNAs was administered 7, 14, and 21 days after the initial transfection. Silencing was validated by RT-qPCR. Cytotoxic effects were observed above 11,000 pmol siRNA and if added with medium supplemented with antibiotics and high concentrations of serum (> 4%). Internal control of lipofectamine alone was used.

### Protein pull-down and Western blot analysis

Proteins lysates were prepared using a lysis buffer provided by the Active Cdc42 Pull-Down and Detection Kit (#16119; Thermo Scientific); 700 μl of each lysate were used for every assay. The three most common members of the Rho-GTPase family are Cdc42, Rac, and Rho. In its active (GTP-bound) state, Rho binds specifically to the Rho-binding domain (RBD) of Rhotekin, and Rac or Cdc42 binds specifically to the p21-binding domain (PBD) of p21-activated protein kinase (PAK) to control downstream signaling cascades. Cdc42-GTP was precipitated by fusion to PBD-PAK1 with glutathione agarose resin as well as Rac, where Rho was precipated by fusion to RBD-Rhotekin. The reaction mixtures were incubated at 4 °C for 1 h in spin cups. The resin was removed and the protein separated on 12% acrylamide gel following the manufacturer’s instructions. GTPγS and GDP were used to generate positive and negative control lysates, respectively. Protein concentrations were determined using the Bradford Reagent (Sigma Aldrich); 20 μg of proteins was loaded in all experiments. SDS-PAGE analysis was performed as previously described by Makdissy et al. [55] on 10% resolving gels. The proteins were transferred onto a PVDF membrane using a Bio-Rad Mini Trans-Blot apparatus and detected using a Protein Detector LumiGLO Western Blot Kit (54–12-50, KPL Laboratories, Gaithersburg, MD, USA) according to the manufacturer’s instructions. The membranes were probed with the diluted primary human antibodies (anti-:) Cdc42-GTP, Cdc42GAP, panRho, p190RhoGAP, panRac, p70RacGAP, albumin, CK-18, AFP, PPARγ, Pref-1, FABP4, nestin, NeuN, O4, GFAP, p-JNK, p-ERK (ERK1/2), p-p38, and p-PKB, p-CREB overnight at 4 °C (all phosphorylated proteins were controlled relative to the total targeted protein). Bound antibodies were detected by incubation with a with horseradish peroxidase-conjugated goat anti-mouse IgG antibody at room temperature. The detection was performed with SuperSignal West Pico Chemiluminescent Substrate (#34080, Thermo scientific) and followed by exposure to x-ray film. The exposure time was 2 s. The images were captured and quantified using a Gel Doc 2000 imaging system and Quantity One software (Bio-Rad). GAPDH was used as an internal control.

### Cell proliferation assay

To estimate the proliferation rate of the cells under different cell culture conditions, doubling time assays were performed. A total of 10^3^ cells/cm^2^ were plated in 12-well tissue culture plates (in triplicate) with 0.5 ml/well of culture medium and incubated at 37 °C, 5% CO_2_, for 0–96 h. At each endpoint, cells were washed once with 1 ml phosphate-buffered saline (PBS) 1×, detached with 0.2 ml trypsin/EDTA, resuspended in 0.5 ml complete culture medium, and counted on a hemocytometer. Results were validated by MTT colorimetric assay (Sigma-Aldrich). Cell doubling (*n*) was determined as following: Cf/C0 = 2^*n*^ (*n* = (logCf – logC0)/log2), where C0 and Cf are the number of cells at time T0 and Tfinal, respectively.

### Adhesion assay

We used 24-well tissue culture plates coated with fibronectin (Millipore PIFB24P05) for adhesion assays; 10^5^ cells were plated in each well, and allowed to adhere for between 10 min and 96 h at 37 °C. Subsequently, the nonadherent cells were carefully removed at each time point and counted. The percentage of adhesion was calculated as a ratio of the number of adherent cells in each sample to the total number of cells added to the coated wells.

### DNA methylation assay

For the determination of global DNA methylation, the Imprint Methylated DNA Quantification Kit (MDQ1, Sigma-Aldrich) was used according to the manufacturer’s instructions. Samples and methylated DNA control were assessed in triplicate.

### Immunohistochemistry (IHC) staining

We adhered 10^4^ cells to a slide by Cytospin® centrifugation and probed with primary antibodies (anti-vimentin, anti-Ki67, and anti-AFP (1/250; Abcam)). IHC was performed on NexEs (benchmark, ROCHES) automate. Images were captured using a Zeiss microscope.

### Dosage of albumin by ELISA

The level of albumin was measured in cell supernatants using an enzyme-linked immunosorbent assay (ELISA) quantification kit (#ab108788, Abcam) in accordance with the manufacturer’s instructions. Briefly, supernatants were first incubated with biotinylated albumin antibody, and then the conjugate, and later with the chromogen substrate. The optical absorbance was measured on the Varioskan™ Multimode Microplate Reader (Thermofisher scientific) at 450 nm.

### Low-density lipoprotein (LDL) uptake assay

The Fluorometric LDL Uptake Assay Kit (#ab204716, Abcam) was used to assess the LDL uptake capacity; 3 × 10^4^ hADSCs and hepatocyte-like cells were incubated with fluorescent-labeled LDL overnight at 37 °C and, after incubation, cells were washed and fluorescence measured on the Varioskan™ LUX Multimode Microplate Reader. Cell culture medium alone and the HepG2 cell line were used as negative and positive controls, respectively. RFU values were calculated from a standard curve following the manufacturer’s instructions.

### Urea production assay

The amount of urea present in the cell lysate was assessed using the colorimetric urea assay kit (#MAK006, Sigma**-**Aldrich) according to the manufacturer’s instructions. The optical absorbance was measured on Varioskan™ LUX Multimode Microplate Reader (Thermofisher scientific) at 570 nm. Human urine served as the positive control.

### CYP activity assay

The cytochrome P450 enzyme activities were determined at day 28 of the Hep-Dif according to Kim et al. [56]. At day 28, the culture medium was supplemented with CYP substrate (CYP-IPA (2 μM)) to measure the enzymatic activities of CYP3A4. Cells were then incubated at 37 °C with 5% CO_2_ in a humidified atmosphere for 1 h. After incubation, 100 μl of the culture medium was transferred to a 96-well opaque white luminometer plate at room temperature and luciferin detection reagent was added to the sample (v:v, 1:1). After 30 min of incubation at room temperature, luminescence was measured using a luminometer. Blank samples without cells were used and corresponding values of luminescence were subtracted from obtained values of the samples. Rifampicin (20 μM, 24 h) was used as a positive inducer. The CYP enzyme activity (Δluminescence/min) = absorbance in induced HLCs – absorbance in basal HLCs.

### Periodic acid-Schiff (PAS) staining

Glycogen storage was evaluated using the PAS staining kit (#395B, Sigma-Aldrich). Cells were fixed with 4% formaldehyde following the manufacturer’s instructions and the HepG2 cell line was used as positive control. Samples were assessed under a light microscope (Nikon).

### Exosome purification

Exosome release was collected from the culture medium of 10^6^ cells and isolated by ultracentrifugation according to the method previously described by Thery et al. [57]. Purified exosomes were fixed and incubated with the specific tetraspanins exosome biomarkers: human anti-CD9 (PE), CD63 (APC), CD81 (PerCP), and isotype control antibodies from MACS (Miltenyi-Biotec). Released exosomes were quantitated by measuring the activity of their specific enzyme, acetylcholinesterase (AChE; MAK119, Sigma-Aldrich). Exosomes fractions were suspended to PBS (1v:4v) and incubated with 5,5′-dithiobis(2-nitrobenzoic acid) (100 μM) and acetylthiocholine (125 μM) in a final volume of 1 ml at 37 °C, and the change in absorbance at 412 nm was determined continuously up to 240 min. The data represent the enzymatic activity at 30 min of incubation at maximum saturation.

### Flow cytometry analysis

For surface marker immunophenotyping, cells were stained with the following conjugated antibodies: anti-CD45-FITC, anti-CD34-PE, anti-CD14-PE, anti-CD73-APC, anti-CD90-FITC, anti-CD105-Vioblue, and relevant isotypes (Miltenyi-Biotec). 7AAD and Annexin V/PI were used to assess cell viability and apoptosis. At least 20,000 events for test samples were acquired. The MACSplex Cytokin12 kit was used for the cytokines analysis. Supernatants were mixed to capture specific beads for each cytokine: granulocyte/macrophage colony-stimulating factor (GM-CSF), interferon (IFN)-α, IFN-γ, interleukin (IL)-2, IL-4, IL-5, IL-6, IL-9, IL-10, IL-12p70, IL-17A, and tumor necrosis factor (TNF)-α. PE-conjugated antibodies were added and incubated for 2 h at room temperature and away from light. After centrifugation, the pellets containing beads were resuspended; flow cytometric acquisition and data analysis were performed by the MACSQuant® Express Mode. Background signals were determined by analyzing beads incubated with the cell culture medium alone. The background signals were subtracted from the signals obtained for beads incubated with supernatants.

### Statistical analysis

All experiments were performed in triplicates. Results are presented as the means ± SEM and have been analyzed for statistical significance (on absolute values) using a Student’s *t* test. Pearson coefficient *R* and its *P* value were calculated for the correlation measures. For all statistical tests, *P* values were two-tailed and the level of significance was set at 0.05.

## Results

### Cdc42 activity increases with age in hADSCs

The screening of Cdc42 activity in undifferentiated hADSCs derived from 61 donors showed an age-dependent significant increase in Cdc42-GTP that correlated significantly with a decrease in Cdc42GAP (Fig. 1). In fact, Cdc42-GTP was positively correlated to the age of the donors contrary to Cdc42GAP (*R* = 0.934 (*P* < 0.0001) and *R* = −0.809 (*P* < 0.0001), respectively (Fig. 1a, b)); a negative correlation was found between Cdc42-GTP and Cdc42GAP (*R* = −0.807 (*P* < 0.0001) (Fig. 1c)) indicating that inhibition of Cdc42GAP is needed to increase the levels of Cdc42-GTP in hADSCs dependently of donor age. No significant correlations were found between Cdc42-GTP and the cell yield/mg of collected adipose tissue (Fig. 1d).

Next, we proceeded to pharmacologically inhibit the Cdc42 activity using the selective Cdc42/Rac1 GTPase inhibitor ML141 [27]. Undifferentiated cells were cultured for 96 h to reach 80% confluence and then treated or not with ML141 (10 μM, 24 h), and the activity of Cdc42 was assessed. ML141 was able to reverse significantly the age-related behavior of cultured undifferentiated hADSCs by decreasing the activity of the Cdc42-GTP binding complexes (Fig. 1e, f) (*R* = 0.640 (*P* < 0.0001) and *R* = 0.631 (*P* < 0.0001) for Cdc42-GTP and Cdc42GAP, respectively). Furthermore, we investigated whether ML-141 effected on the other Rho family GTPases Rac and Rho. In fact, the three most common members of the Rho family GTPases are Cdc42, Rac1, and RhoA. Rho-GTP and Rac-GTP active proteins were studied using pan-antibodies directed against RhoA/B/C and Rac1/2/3. Without ML141 treatment, we observed significant positive correlations between the age of the donor and Rho-GTP and Rac-GTP (Fig. 1g, h). Importantly, in the aged group, ML141 efficiently blocked the Cdc42 association with GTPγS and PAK-PBD, and decreased GTP-Cdc42 (−87.7%, *P* < 0.0001) and GTP-Rac1 (−41.0%, *P* < 0.001) content in undifferentiated hADSCs. However, a moderate and nonsignificant decrease was observed in the case of GTP-Rho (−21.6.0%, *P* = 0.064). As expected, RacGAP and RhoGAP increased (data not shown).

Then, two groups of donors designated for their age as young (*n* = 19, age < 26 years (mean age = 23.8 ± 0.4)) and aged (*n* = 20, age > 50 years (mean age = 57.6 ± 0.9)) based on their Cdc42-GTP activity as shown in Fig. 1a were selected (Additional file 2: Table S2) for the followed parts of this study. The impact of ML141 on the proliferation and differentiation of hADSCs derived from these two groups of donors was studied.

### Effects of pharmacological targeting of Cdc42 activity by ML141 on the proliferative, adherence, and apoptosis characteristics of hADSCs

**Fig. 2:**
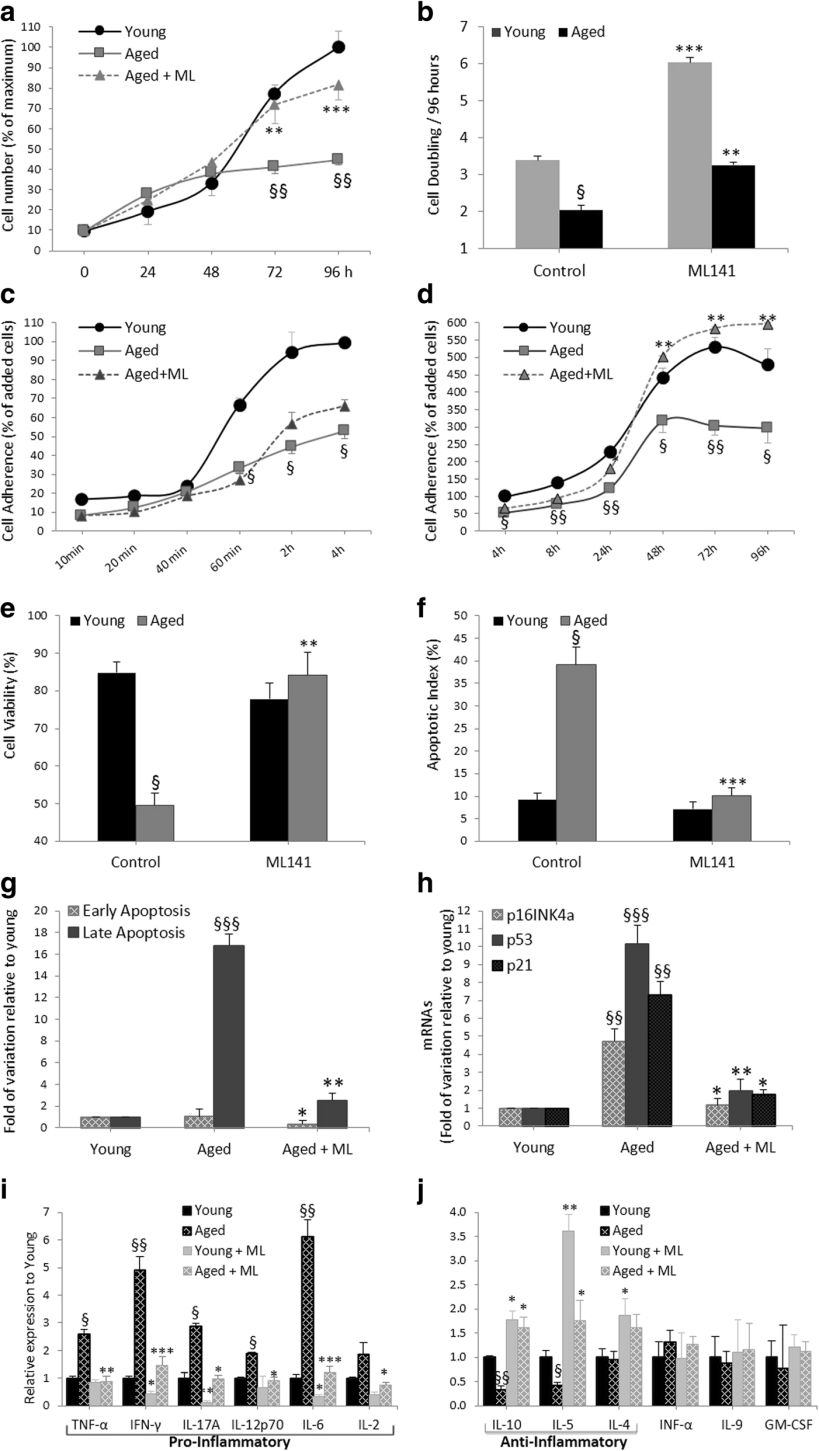
Pharmacological targeting of Cdc42 activity by ML141 (ML) on the yield, growth, adherence, apoptosis, and immunomodulatory characteristics of hADSCs. Cells derived from young and aged subjects were cultured for 96 h. Cells were treated with or without ML141 (10 μM, 24 h). Proliferative potential as indicated by cell number (**a**) and time for population doubling (**b**). Kinetics of hADSC adherence as evaluated by the number of adhered cells and expressed as the percentage of applied cells (**c,d**). Cell viability (**e**) and apoptotic index (**f**) as assessed by labeled cells with Annexin V/propidium iodide (PI)/7AAD. The cell viability and apoptotic index were expressed respectively as the percentage of Annexin V^(−)^/PI^(−)^/7AAD^(−)^ or Annexin V^(+)^ cells divided by total cells. **g** Early from late-apoptotic cells were identified as Annexin V^(+)^/PI^(−)^/7AAD^(−)^ vs Annexin V^(+)^/PI^(+)^/7AAD^(+)^, respectively. **h** Real-time quantitative RT-PCR determination of p16^INK4a^, p53, and p21 mRNA levels. The results are expressed as fold variations ± SEM over the young group (*R*) after normalization to β-actin. **i,j** Cell culture supernatants from undifferentiated hADSCs were collected and undiluted samples were analyzed for the detection of cytokines as indicated in the Methods. Three to five measures were realized by group and results are the mean ± SEM presented in fold of variation relative to young. The cytokine limits of detection (pg/ml) were: tumor necrosis factor (TNF)-α (2.17), interferon (IFN)-γ 6.26, IFN-α (10.4), granulocyte/macrophage colony-stimulating factor (GM-CSF) (0.20), interleukin (IL)-2 (1.52), IL-4 (34.2), IL-5 (0.27), IL-6 (0.077), IL-9 (32.7), IL-10 (2.76), IL-12 (3.44), and IL-17A (0.7). The results represent individual expression per subject (young (*n* = 19), aged (*n* = 20) and aged+ML141 (*n* = 20)) and are the means of two independent experiments performed in triplicate. ^§^*P < 0.05, ^§§^**P < 0.01, ^§§§^***P < 0.001; ^§^aged versus young and *aged treated with ML141 versus untreated

We investigated the impact of ML141 on the yield, growth, adherence, and apoptosis of undifferentiated hADSCs between the aged and young groups. Cells derived from the aged group showed significant decreases in the cell proliferation, doubling, adherence, and viability, with a concomitant increase in the apoptotic index (Fig. 2). Compared to the nontreated control aged hADSC group, ML141 significantly improved the cell growth and doubling time in aged group-treated hADSCs (Fig. 2a, b). The adherence feature of the cells was markedly decreased in the aged group, and ML141 significantly improved the adherence, especially at 48 h (Fig. 2c, d). Cell viability was markedly reduced in the aged group accompanied by an elevated apoptotic index, where ML141 significantly reversed these effects (Fig. 2e, f). Importantly, aged-derived ADSCs were mostly affected at late apoptosis (Fig 2g) and this was improved by ML141 treatment. The observed variations in the apoptotic index can be explained by the expressions of the apoptotic genes p16, p53, and p21, which were significantly higher in the aged group, and ML141 was able to reverse significantly the expression of p16, p53, and p21 mRNAs in the aged group-treated hADSCs (Fig 2h).

### Impact of Cdc42 inhibition on the immunomodulatory ability of undifferentiated MSCs

We evaluated the impact of ML141 on the immunomodulatory behavior of undifferentiated hADSCs. The analyses of the cytokine profiles determined by flow cytometry on the collected supernatants of undifferentiated ADSCs showed that the levels of proinflammatory cytokines (TNF-α/IFN-γ/IL-17A/IL-12p70/IL-6/IL-2) were elevated in the aged group compared with the young group (Fig. 2i), contrary to the levels of the anti-inflammatory cytokines (IL-10 and IL-5) which were decreased (Fig. 2j). The levels of IL-4/IL-9/IFN-α/GM-CSF were significantly unchanged. Treatment of the aged group-derived cells with ML141 significantly reversed the pattern observed in cells from the aged donors and demonstrates the evident impact of ML141 having immunomodulatory effects, thus reducing the levels of proinflammatory cytokines and improving the levels of the anti-inflammatory cytokines.

### Phenotypic characterization of differentiated cells into hepatocyte-like cells: impact of ML141

**Fig. 3:**
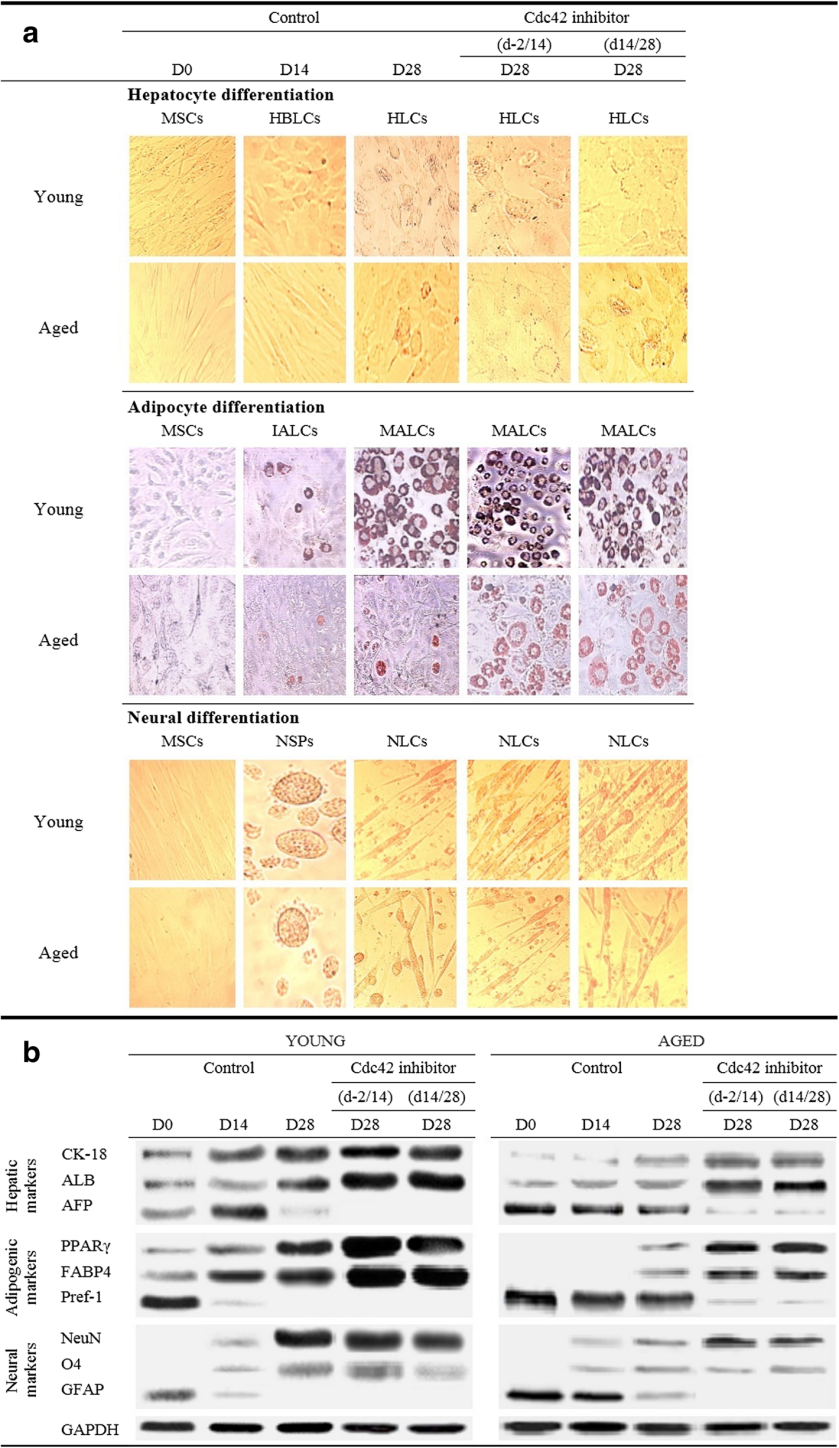
Impact of ML141 on hepatic, adipogenic, and neurogenic differentiation of young and aged-derived hADSCs. hADSCs were isolated from young or aged subjects and subjected to hepatocyte/adipocyte/neural differentiation as indicated in the Methods for 28 days. Cells were collected at day 0 (MSCs) at the moment of induction of the differentiation, and days 14 and 28 of the differentiation. Cells were incubated with or without 10 μM ML141 following two protocols of treatment: from day −2 to day 14 of the differentiation (d-2/14) and from day 14 to day 28 of the differentiation (d14/28). The effect of ML141 was evaluated at day 28 of the differentiation. **a** Representative images of morphological cell changes from: undifferentiated MSCs at D0, hepatoblast-like cells (HBLCs) or immature adipocyte-like cells (IALCs) or neurospheres (NSPs) at D14, hepatocyte-like cells (HLCs) or mature adipocyte-like cells (MALCs) or neural-like cells (NLCs) at D28. Cells from adipocyte differentiation were stained with oil-red O. **b** Hepatic/adipogenic/neurogenic marker expression: cell lysates (80–150 μg of protein) were separated by SDS-PAGE and immunoblotted with antibodies raised against cytokeratin (CK)-18, albumin (ALB), alpha fetoprotein (AFP), peroxisome proliferator activated receptor (PPAR)γ, FABP4, Pref-1, NeuN, O4, GFAP, and GAPDH. Protein expression profiling was determined during differentiation at D0/14/28; representative blots are shown

The multipotency of hADSCs derived from young and aged donors was confirmed by inducing trilineage differentiation of hADSCs toward mesodermal (adipocyte, Adp-Dif), endodermal (hepatocyte, Hep-Dif), and ectodermal (neural, Neu-Dif) lineage during 28 days (Fig. 3). hADSCs gradually changed from fibroblast-like cells to a broad, flattened shape, and the majority of the cells were changed into hepatocyte-like morphology at D28 of the Hep*-*Dif. Immature adipocyte-like cells (IALCs) and full mature adipocyte-like cells (MALCs) were shown at D14 and D28 of the Adp-Dif as assessed by Oil Red O staining assay. Neurospheres (NSPs) and neural-like morphology were observed at D14 and D28 of the Neu-Dif. Adherence and viability (7AAD staining vs Annexin V/PI) were significant and above 89% in these two groups.

In comparison with the young group, the aged-derived cells showed lower potentials to differentiate into hepatocyte-, adipocyte-, and neural-like cells (Fig. 3a). The differentiation potentials were confirmed (Fig. 3b) by the expression of the following specific proteins: 1) CK-18 and albumin (ALB) (specific markers of hepatogenesis) increased during Hep-Dif, whereas AFP increased for 14 days and then decreased; 2) PPARγ and FABP-4 (specific markers of adipogenesis) increased during Adp-Dif while Pref-1 (a known inhibitor of the Adp-Dif) decreased; and 3) NeuN and O4 (specific markers of neuron and oligodendrocytes-like cells, respectively) increased in contrast to GFAP (a specific marker of astrocyte-like cells) which decreased. Therefore, our results demonstrated that hepatocyte/adipocyte/neural differentiation of aged-derived hADSCs were markedly impaired. Importantly, despite decreased adipogenesis induction, aged hADSCs showed a maintained potential towards adipogenesis (low) in comparison with osteogenesis and chondrogenesis which were more markedly inhibited. In fact, aged hADSCs showed high and significant decline in the potential for osteogenesis and chondrogenesis as determined by the low mRNA and protein expression of alkaline phosphatase/osteocalcin and collagen II/aggrecan, respectively (data not shown). The loss of the adipogenesis potential was confirmed (as shown in Fig. 3b) by the fact that aged hADSCs had low levels of PPARγ and FABP at day 28 of the adipogenic differentiation but maintained high levels of Pref-1.

The impact of ML141 was also assessed on hADSC differentiation. hADSCs were divided as follows: young, aged, and ML141-treated aged groups for 48 h (from D–2 to D0), 16 days (from D–2 to D14), 14 days (from D14 to D28), or 30 days (from D–2 to D28). Progressively, we ruled out the 48-h and 30-day treatment groups due to the lack of significant improvements (D–2/0), or aggressive cell apoptosis (D–2/28) (data not shown). Thus, we proceeded to treat the cells with ML141 during the early differentiation (D–2/14) or maturation (D14/28) phases. ML141 improved the potentials of differentiation (as indicated in Fig. 3).

In addition, we immunophenotyped the cells at days 0, 14, and 28 of the Hep-Dif; all hADSCs were negative for the hematopoietic stem cell markers CD14, CD34, CD45, and HLA-DR, but were positive for the specific MSC markers CD90, CD105, and CD73 (Table 1). A decline in the mesenchymal phenotype was observed in the young group indicating a transition to a differentiated phenotype (80–83% of the non-MSC phenotype at D28); however, a moderate potential of differentiation was observed in the aged nontreated group (30–36%). In addition, at day 28 cells from the ML141-treated groups showed higher potentials for differentiation as observed by significant additional declines in the levels of expression of the MSC markers, and these improvements were more pronounced in the ML141-treated young group compared with the ML141-treated aged group (80–93% versus 55–70% of the non-MSCs phenotype at D28, respectively) (Table 1).

**Table 1:** Immunophenotyping of hADSCs derived from young and aged subjects and differentiated into hepatoblast- and hepatocyte-like cells

	(−) ML141				(+) ML141 (d–2/14)	(+) ML141 (d14/28)
	D0	D7	D14	D28	D28	D28
Differentiation of hADSCs derived from young subjects						
HLA-DR	2.03 ± 0.53	0.02 ± 0.11	2.55 ± 0.11	0.51 ± 0.92		
CD14^+^	0.12 ± 0.40	0.14 ± 0.24	0.15 ± 0.38	0.08 ± 0.32		
CD34^+^	1.17 ± 1.88	6.03 ± 0.52	3.20 ± 0.68	1.12 ± 0.44		
CD45^+^	0.06 ± 0.43	0.07 ± 0.98	0.05 ± 0.12	0.26 ± 0.75		
CD73^+^	88.61 ± 3.92	72.30 ± 10.39*	37.33 ± 2.05**	19.14 ± 1.62***	16.44 ± 5.23**	21.36 ± 3.98**
CD90^+^	90.96 ± 6.80	74.43 ± 3.76*	36.41 ± 2.16***	17.09 ± 0.84***	10.38 ± 3.41***	7.33 ± 4.26***
CD105^+^	91.09 ± 7.68	53.07 ± 1.24*	40.25 ± 6.43***	20.72 ± 3.51***	14.25 ± 4.12***	15.13 ± 5.22***
Differentiation of hADSCs derived from aged subjects						
HLA-DR	1.44 ± 0.37	0.33 ± 0.10	1.50 ± 0.42	1.61 ± 0.86		
CD14^+^	0.11 ± 0.10	0.91 ± 0.82	0.11 ± 0.41	0.42 ± 0.13		
CD34^+^	1.43 ± 0.24	1.80 ± 0.39	1.51 ± 0.80	0.98 ± 1.10		
CD45^+^	0.02 ± 0.23	0.08 ± 0.34	0.03 ± 0.21	0.12 ± 0.33		
CD73^+^	94.68 ± 3.58	85.07 ± 1.04	80.96 ± 4.96*^§§^	70.46 ± 2.29*^§§^	35.57 ± 0.71*^#^	29.43 ± 3.16*^#^
CD90^+^	91.41 ± 6.80	80.23 ± 3.76	75.95 ± 2.11*^§§^	70.01 ± 4.08*^§§^	31.43 ± 1.60*^#^	35.95 ± 1.42*^#^
CD105^+^	92.88 ± 3.09	71.40 ± 2.36*^§^	73.03 ± 2.30*^§§^	64.92 ± 3.15*^§§^	34.63 ± 3.09*^#^	45.57 ± 2.81*^#^

Human adipose-derived mesenchymal stem cells (hADSCs) isolated from young and aged donors were cultured and differentiated as described in the Methods for 28 days. Cells were incubated with or without 10 μM ML141 following two protocols of treatment: from day −2 to day 14 of the differentiation (d-2/14) and from day 14 to day 28 of the differentiation (d14/28). Cells were collected at day 0 (MSCs) at the moment of induction of the differentiation, and days 7, 14, and 28 of the differentiation (hepatoblast-like cells and hepatocyte-like cells, respectively). Cells were labeled with fluorescence-coupled antibodies against HLA-DR, CD14, CD34, CD45, CD73, CD90, and CD105, and analyzed using a MACSQuant flow analyzer as indicated in the Methods. The results are expressed as the percentage of cell surface marker per total number of cells, and are the mean ± SEM of 19 and 20 subjects each performed in duplicate in young and aged groups, respectively. Effect of ML141 was evaluated at day 28 of the differentiation. *P < 0.05, **P < 0.01, ***P < 0.005, D14/D28 versus D0; ^§^*P* < 0.05, ^§§^*P* < 0.01, aged versus young; ^#^*P* < 0.01, treated with ML141 versus untreated in the same group

### Impact of ML141 on the gene expression profile during Hep-Dif

**Fig. 4:**
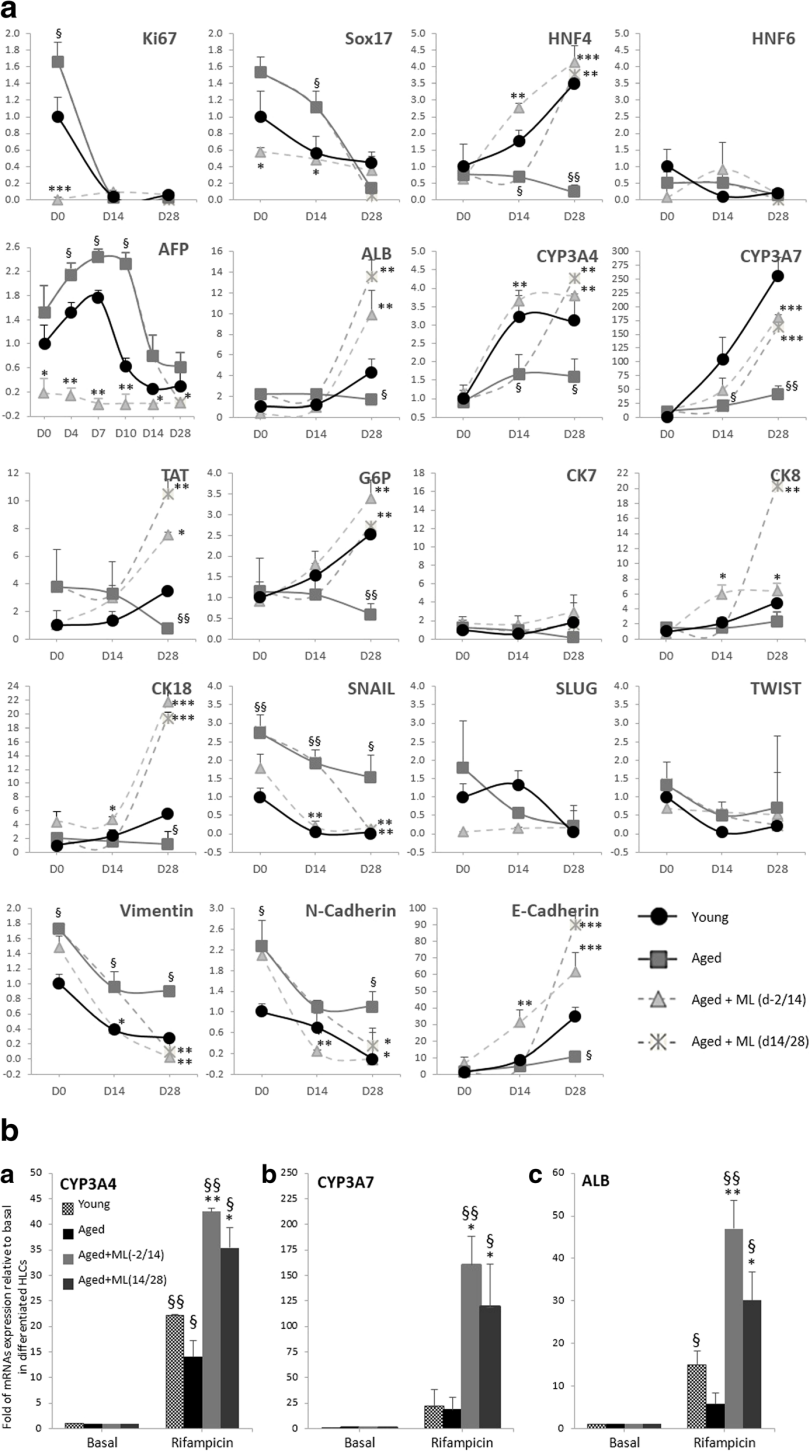
Impact of pharmacological targeting of Cdc42 activity by ML141 (ML) on the gene expression profile during Hep-Dif. hADSCs were isolated from young and aged subjects and were induced to Hep-Dif for 28 days with or without ML141 (10 μM) for the indicated time of incubation. RNAs were collected at D0/14/28 and mRNA levels of the studied genes were quantified by RT-qPCR. **a** Gene expression profile of hepatic and mesenchymal-to-epithelial transition markers as well as epigenetic markers (the DNA methyltransferases, DNMTs). **b** Rifampicin responsiveness of hepatocyte-like cells: RNAs were collected at day 28 of the differentiation after cell treatment with rifampicin (20 μM, 24 h). Expression levels of CYP3A4, CYP3A7, and albumin (ALB) genes are shown. The results are expressed as fold variation relative to controls (without rifampicin) after normalization to GAPDH, and are the mean ± SEM of two independent experiments performed twice, each in duplicate. D0 = day of induction of the differentiation. d-2/14, d14/28: cells were treated with ML141 from day −2 to day 14, or day 14 to day 28, respectively. ^§^*P < 0.05, ^§§^**P < 0.01, ***P < 0.001; ^§^aged versus young or rifampicin versus basal and *aged treated with ML141 versus untreated

The expression of several genes which play key roles in Hep-Dif was evaluated (Fig. 4a). Compared with the young group, our results showed that, at day 14 postinduction, differentiating cells had already lost their Ki67 expression indicating a hepatogenic commitment; Sox17, the specific marker of the endodermic lineage, was shown to be highly expressed in the aged group and ML141 significantly reduced its expression. To the contrary, it increased the expression of HNF4α, the master gene of the hepatogenic lineage, to levels similar to those seen in the young group; HNF6 remained unaffected. AFP, the hepatoblast fetal gene, showed a significant increase during the early phase (D0–14) followed by a marked drop in its expression in contrast to ALB which was significantly increased during Hep-Dif by ML141. CYP3A4 (premature form) and CYP3A7 (natal hepatocyte form) increased during differentiation in both the young and ML141-treated groups. We next checked the expression of genes responsible for the metabolic activity. TAT and glucose-6-phosphate (G6P) expressions were significantly reduced in the aged compared with the young group, and reversed by ML141 treatment. In addition, ML141 significantly induced the expression of hepatic functional genes, the epithelial cytokeratin CK-8 and CK-18, but not CK-7.

The mesenchymal-epithelial transition (MET) gene levels reflected a variable and age-dependent pattern in the different groups: SNAIL, Vimentin, and N-cadherin decreased during the differentiation with higher levels in the aged untreated group, in contrast to E-cadherin which increased; ML141 reversed the situation in the aged group to a young-like state. SLUG and TWIST did not show a significant variation in all differentiated groups.

To assess the responsiveness of the differentiated HLCs, cells were treated with rifampicin [58] for 24 h at day 27, and the mRNA expression of CYP450 enzymes, ALB, TAT, and G6P were determined (Fig. 4b). HLCs following rifampicin induction displayed significant increases in the expression of Cyp3A4, Cyp3A7, and ALB in the ML141-treated groups. TAT and G6P expressions were unchanged (data not shown). These results indicate that ML141 treatment in the aged group had the ability to reverse the expression of specific hepatogenesis genes.

**Fig. 5:**
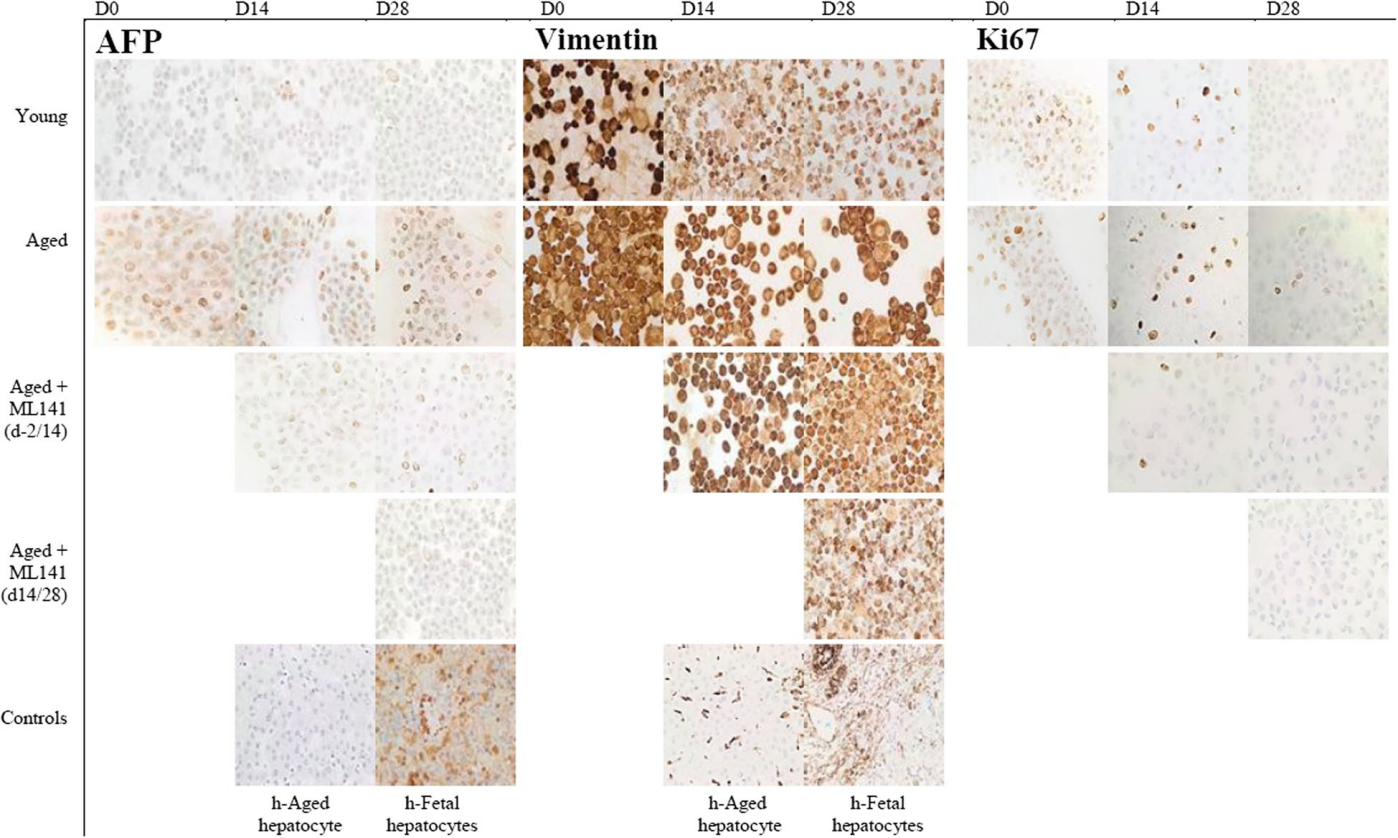
Extinction of alpha fetoprotein (AFP), Vimentin, and Ki67 from hepatocyte-like cells derived from aged ADSCs treated with ML141. Cells derived from young and aged groups were differentiated and treated with or without ML141 as indicated in Fig. 4 and evaluated for AFP (fetal hepatoblast marker), Vimentin (EMT marker), and Ki67 (proliferation marker) by immunohistochemistry (IHC) as described in the Methods. Cells were examined microscopically and phase-contrast images were captured. Cultures of primary human (h) fetal and adult hepatocytes were assessed as positive controls and negative controls, respectively

The mRNA results for Vimentin, Ki67, and AFP were confirmed by immunohistochemistry (Fig. 5). A high expression of Vimentin was seen in the untreated cells from the aged group and this was inhibited by ML141 indicating an epithelial transition of hADSCs. Ki67 protein expression disappeared during differentiation and ML141 maintained its absence in HLCs. Concomitantly, the expression of AFP protein persisted in nontreated aged hADSCs and disappeared after ML141 treatment. Vimentin expression displayed similar results. These results are in favor of a hepatogenic commitment and maturation of treated ADSCs.

### ML141 restores hADSC function during Hep-Dif

**Fig. 6:**
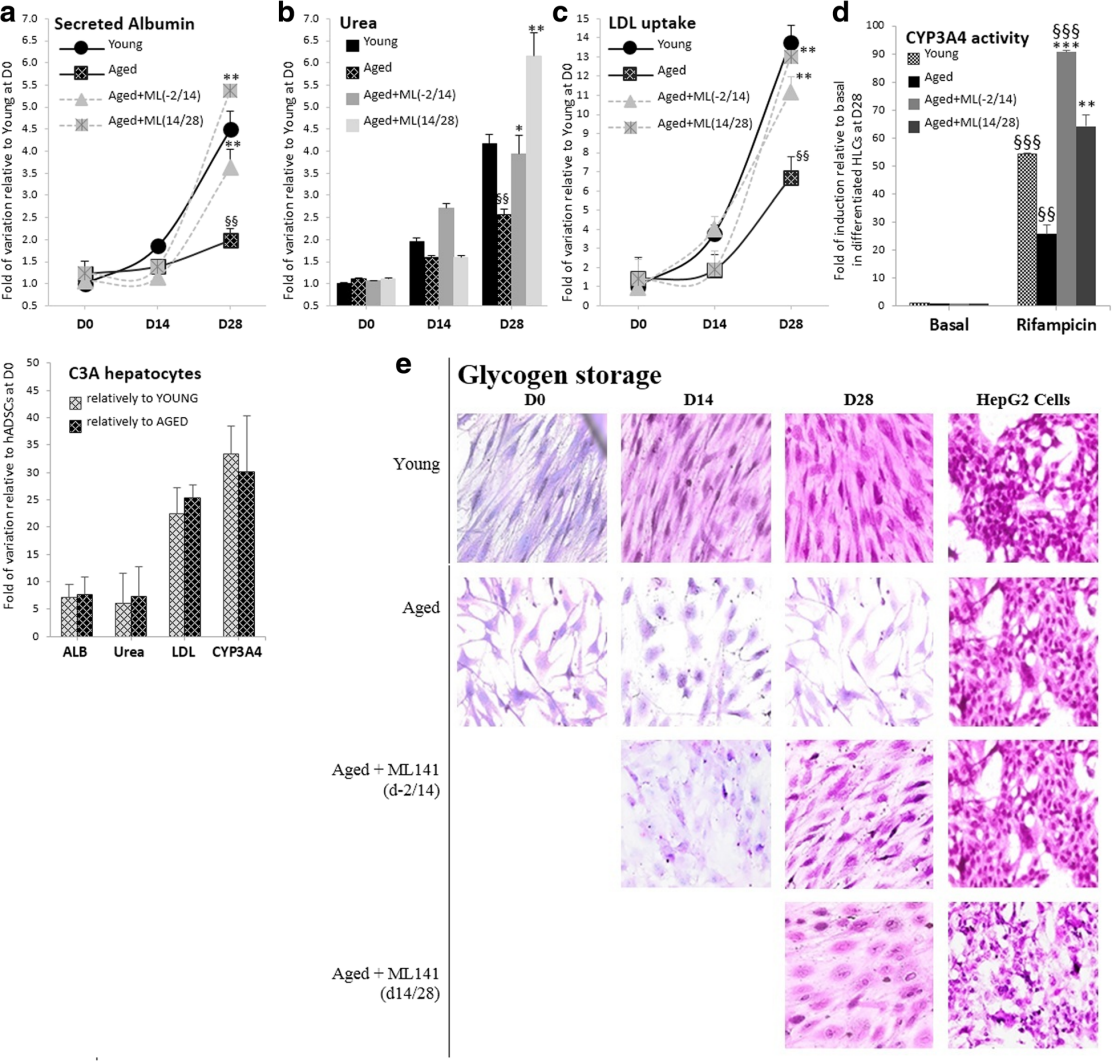
Pharmacological targeting of Cdc42 activity by ML141 (ML) induces rifampicin responsiveness and restores cell function. hADSCs derived from young and aged subjects were differentiated and treated with or without ML141 as indicated in Fig. 4. **a–c** Secreted albumin, urea production, and low-density lipoprotein (LDL) uptake: supernatants of cultured cells and the lysates were collected at D0/D14/D28 of the differentiation for the quantification of albumin, urea, and LDL uptake as indicated in the Methods. Results are expressed per ng/ml (for the production of albumin and urea) and RFU (fluorescent LDL uptake) and are presented as fold variation relative to young at day D0, and are the mean ± SEM of several measures (5, 6 and 10, respectively). **d** Cytochrome P450 (CYP3A4) activity at day 28 of the Hep-Dif after induction with rifampicin (20 μM, 24 h). The results are expressed as fold variation relative to basal (without rifampicin), and are the mean ± SEM of three independent experiments performed in duplicate. **e** hepatocyte-like cells derived from aged ADSCs treated with ML141 exhibit hepatic-specific function of glycogen storage. Cells were evaluated for glycogen storage capacity (pink color) using periodic acid-Schiff (PAS) staining as described in the Methods. Cells were examined microscopically and phase-contrast images were captured. Cultures of HepG2 cells were assessed as positive controls. ^§^*P < 0.05, ^§§^**P < 0.01; ^§^rifampicin versus controls or aged versus young and *aged treated with ML141 versus untreated

To test the functionality of the obtained HLCs, we quantified the secretion of albumin, production of urea, uptake of LDL, CYP3A4 enzyme activity, and glycogen storage (Fig. 6). HLCs derived from the young group produced high levels of albumin and urea, as well as increased levels of LDL uptake (Fig. 6a); the levels increased significantly with the differentiation. However, HLCs derived from the aged group showed lower levels of albumin, urea, and LDL uptake. ML141 treatment improved the tested functions in the aged group. The cytochrome P450 activity as determined by measuring the activity of CYP3A4 enzyme after induction with rifampicin was decreased in the aged-derived hADSCs in comparison with the young group, and ML141 treatment reversed the situation (Fig. 6d). Furthermore, the storage of glycogen as evaluated by PAS staining (Fig. 6e) confirmed the HLC functionality; the morphology of the cells changed from being long-spindled shaped at D0 to short-spindled shaped at D14, and to large-sized, polygonal shaped HLCs at D28. More importantly, cells stored glycogen in the young and ML141-treated groups. In fact, ML141-treated cells retained high levels of purple stain, similar to the cells derived from young donors and HepG2.

### The mechanism of action of ML141 in inducing Hep-Dif of hADSCs

#### Attenuation of Wnt3a/β-catenin

**Fig. 7:**
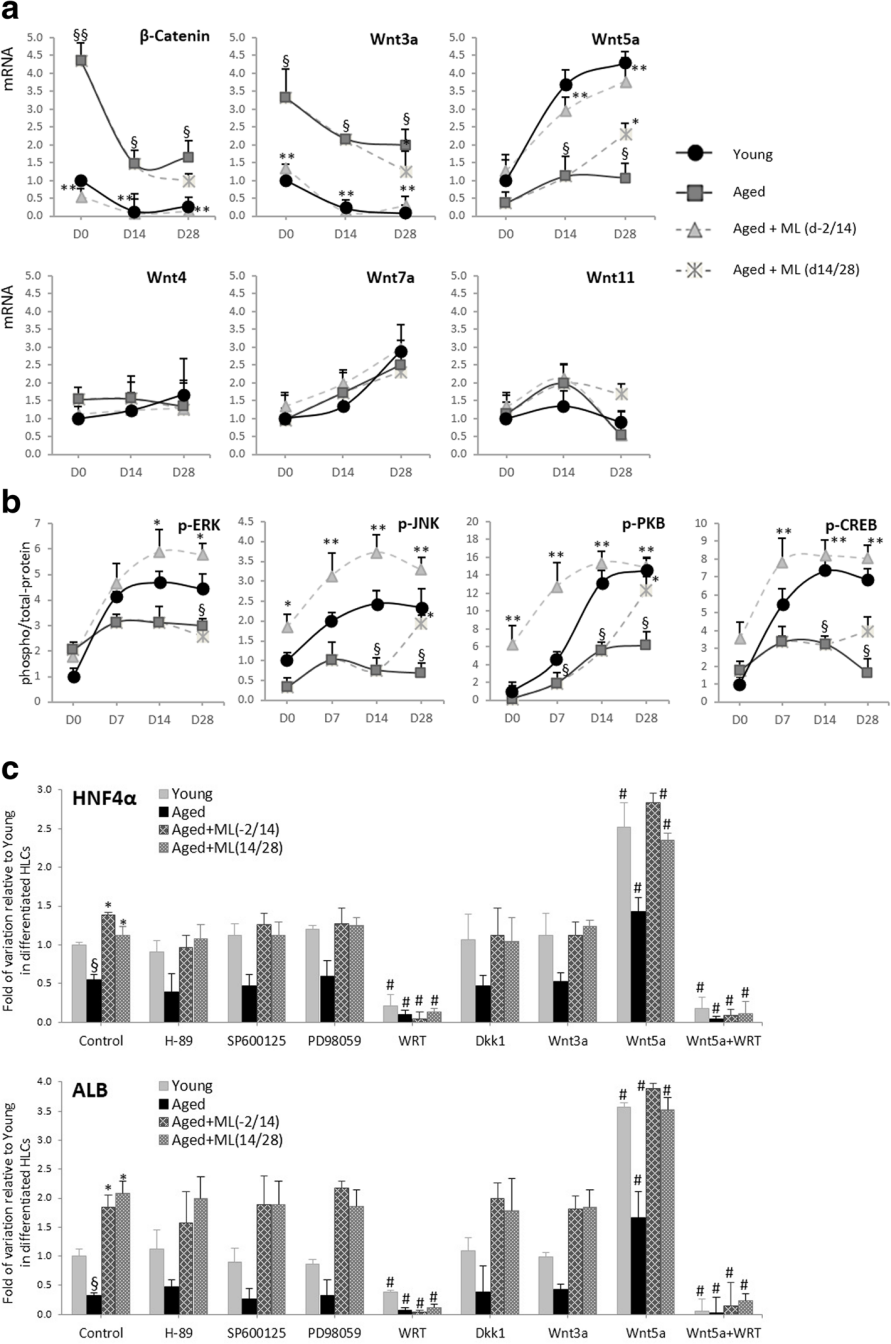
ML141 (ML) impact on hepatocyte differentiation is dependent of PI3K and Wnt5a signaling. hADSCs were induced to Hep-Dif for 28 days with or without ML141 (10 μM) for the indicated time of incubation (d-2/14 or d14/28). Cells were treated 24 h before adding ML141, and maintained with ML141, with: 1) inhibitors of PKA (H-89, 5μM), JNK (SP600125, 10μM), ERK (PD98059, 50μM), and PI3K (Wortmannin, 10μM); or 2) Wnt-antagonist Dkk1 (200 ng/ml, 24 h), Wnt3a (50 ng/ml, 24 h), and Wnt5a (100 ng/ml, 24 h). **a** mRNA expression of Wnt(s) and β-catenin expressed as fold variation relative to young at D0 after normalization to GAPDH. **b** Cell lysates (80–150 μg of protein) were separated by SDS-PAGE and immunoblotted with antibodies raised against phospho and total ERK/JNK/PKB/CREB. Protein expression profiling was determined during differentiation at D0/14/28 and results are expressed as fold variation of phospho/total levels relative to young at D0 after normalization to GAPDH. **c** Impact of H-89/SP/PD/WRT/Dkk1/Wnt3a/Wnt5a on the mRNA expression of the hepatic markers hepatocyte nuclear factor (HNF)4 and albumin (ALB) at D28 (hepatocyte-like cells; HLCs): results are expressed as fold variation relative to young untreated cells. Results are the mean ± SEM of three independent experiments performed in duplicate realized on 19 (young) and 20 (aged, aged+ML141) subjects. ^§^*^#^*P* < 0.05, ^§§^**^##^ *P* < 0.01; ^§^aged versus young, *aged treated with ML141 versus control, and ^#^WRT or Wnt5a-treated cells versus control. WRT Wortmannin

Since the Wnt signaling pathway had been associated with hepatocyte differentiation and that, through the Wnt/β-catenin pathway inhibition, it is possible to promote hepatocyte differentiation [35–38], we sought to determine whether this pathway was indeed affected by ML141. The expression levels of β-catenin and different Wnt(s) were evaluated during Hep-Dif (Fig. 7a). We observed that Wnt3a and β-catenin significantly decreased with the Hep-Dif, in contrast to Wnt5a; however, no significant variations were observed for Wnt4, Wnt7a, or Wnt11. Importantly, compared with the young-derived cells, Wnt3a and β-catenin were highly expressed in aged-derived cells in contrast to Wnt5a. Interestingly, ML141 treatment and more specifically the D−2/14 study significantly reversed the levels of Wnt3a/β-catenin/Wnt5a to nearly the levels obtained in the young group. Our data suggest that extinction of Wnt3a/β-catenin signaling in parallel with induction of Wnt5a may be required for hepatocyte differentiation, and this is affected by ML141 treatment.

#### Induction of PI3K and MAPK signaling pathways

The PI3K/PKB and MAPK signaling pathways have been shown to be involved in hepatic differentiation [30, 31]. To investigate how ML141 impacts these pathways during Hep-Dif, we assessed the effect of ML141 on the activation of the PI3K/PKB and MAPK pathways (Fig. 7b). ML141 positively regulated the phosphorylation of ERK/JNK but not p38 in the MAPK pathway, and more importantly the phosphorylation of PKB in the PI3K pathway. In addition, we assessed whether ML141 affects the activation of cAMP response element-binding protein (CREB), a common target of these pathways; we found that hADSC exposure to ML141 resulted in a marked increase in phospho/total-CREB during Hep-Dif*.* These data indicate that ML141 rescued the levels of the studied phosphorylated proteins of the aged group.

#### PI3K and Wnt5a are required for the ML141-induced Hep-Dif of hADSCs

To further investigate whether PI3K/MAPK/Wnt signaling are required for the ML141-induced hepatocyte differentiation, cells were treated 24 h before adding ML141 and maintained throughout the ML141 incubation with: 1) inhibitors of protein kinase A (PKA) (H-89), JNK (SP600125), ERK (PD98059), and PI3K (Wortmannin (WRT)); or (2) Wnt-antagonist (Dkk1), Wnt3a, or Wnt5a. The expression of HNF4α and ALB mRNAs were assessed at day 28 of the Hep-Dif (Fig. 7c)*.* Surprisingly, none of the PKA/JNK/ERK inhibitors or Wnt3a/DKK1 had any significant impact on the expression of ALB and HNF4α in contrast to WRT and Wnt5a. In fact, the expression of ALB and HNF4α was dramatically decreased by the PI3K inhibitor and markedly increased by Wnt5a. The expression was completely abolished when the cells were treated with combined Wortmannin and Wnt5a, indicating the involvement of a major mechanism of PI3K.

#### Involvement of miR-122 and impact on the exosome release

Hepatocyte differentiation is controlled by a liver-enriched transcription factor (LETFs) network, where miR-122—a direct target of LETF hepatocyte nuclear factor (HNF)—is the most common miRNA in the adult liver and a crucial factor in hepatocyte differentiation [43, 45, 48]. Overexpression of miR-122 promotes Hep-Dif through a miR-122/HNF4α-positive feedback loop [49, 50], and mechanistic studies using inhibitors of PI3K/PKB significantly suppressed the expression of miR-122 levels [51]. We thought it interesting to investigate whether: 1) miR-122 is involved in the ML141-induced Hep-Dif of hADSCs; 2) whether PI3K/MAPK and/or Wnt(s) are involved in the regulation of miR-122; and 3) whether cell functionality is dependent on miR-122.

**Fig. 8:**
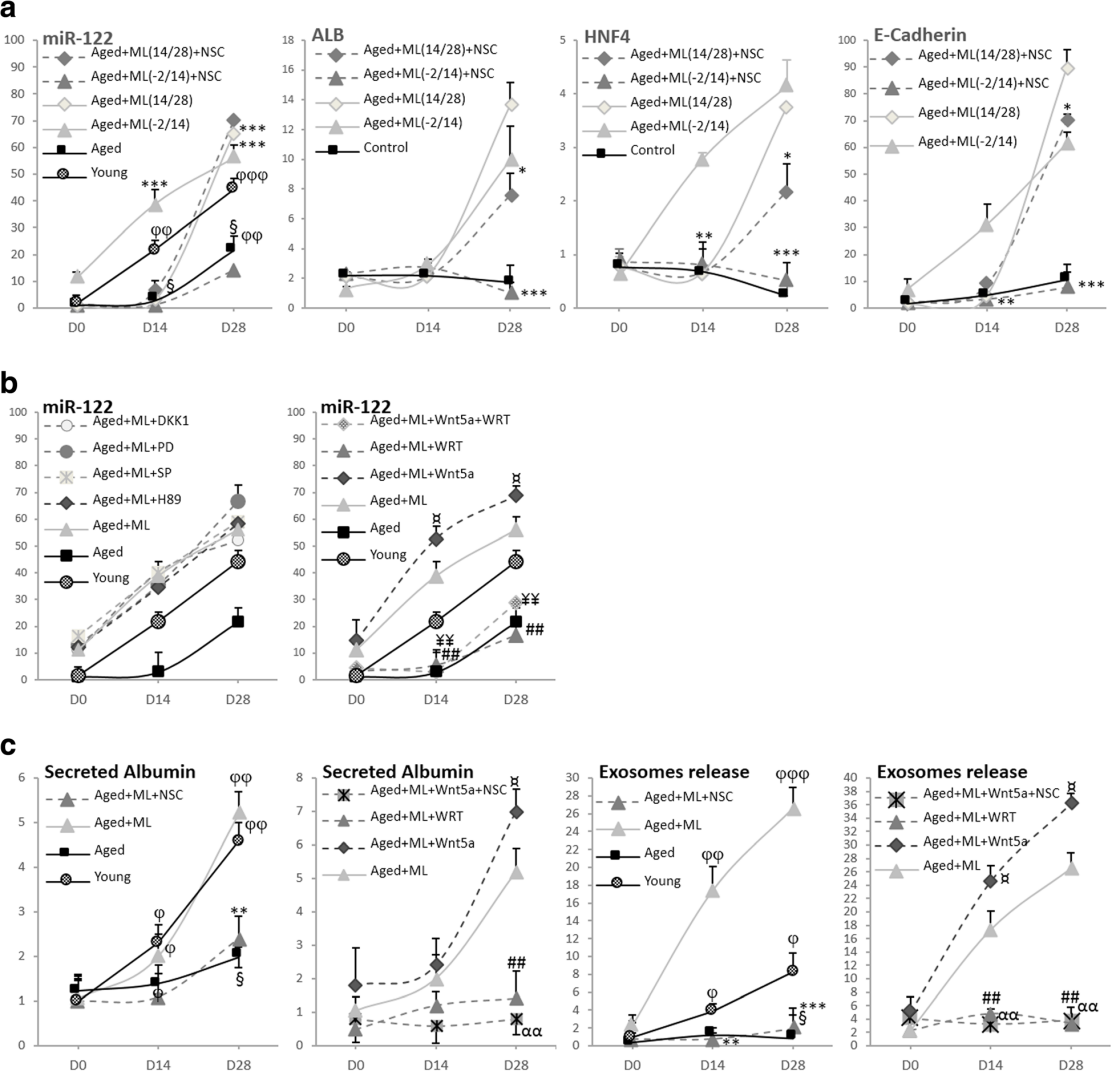
miR-122 selective inhibitor (NSC5476 (NSC)) abolished the effects of ML141 (ML). hADSCs were induced to Hep-Dif for 28 days with or without ML141 (10 μM) for the indicated time of incubation (D-2/14 or D14/28). Cells were incubated as indicated in Fig. 7 (with H-89, SP, PD, WRT, Wnt5a) and with or without NSC5476 (NSC, 5 μM) 24 h before adding ML141 and maintained in parallel to ML141. **a** RNAs were collected at D0/14/28 and mRNA levels of hepatocyte nuclear factor (HNF)4α/albumin (ALB)/E-cadherin/miR-122 genes were determined by RT-qPCR: the results are expressed as fold variation relative to D0 (or young at D0) after normalization to GAPDH. **b** Effects of H-89, SP, PD, WRT, Wnt5a, and NSC on the expression of miR-122 mRNA on the ML(−2/14) treated group. **c** Effects of Wnt5a, WRT, and NSC on the levels of secreted albumin and released exosomes on the ML141(−2/14) treated group. ^¥^*P* < 0.05, aged+ML(14/28) + NSC versus same group without NSC. The results are expressed as fold variation relative to D0 (or young at D0). Results are the mean ± SEM of three independent experiments performed in duplicate realized on 19 (young) and 20 (aged, aged+ML) subjects. ^φ§^*^¤^*P* < 0.05, ^φφ§§^**^## ¥¥αα^*P* < 0.01, ^φφφ§§§^***P < 0.001. ^φ^D14/D28 versus D0, ^§^aged versus young, *aged+ML + NSC versus aged+ML, ^¤^aged+ML + Wnt5a versus aged+ML, ^#^aged+ML + WRT versus aged+ML, ^¥^aged+ML + Wnt5a + WRT versus aged+ML, and ^α^aged + ML + Wnt5a + NSC versus aged+ML. PD PD98059, SP SP600125, WRT Wortmannin

First, the expression of miR-122, HNF4α, albumin, and E-cadherin mRNAs was evaluated during the differentiation (Fig. 8a). Cells were treated with or without the miR-122 selective inhibitor (NSC5476 (NSC)) for 24 h before adding ML141 and maintained throughout the ML141 incubation. As expected, the expression of miR-122 increased significantly during the Hep-Dif, reaching approximately a 44-fold increase relative to the expression of miR-122 at D28 versus 21-fold at D0 (young-hADSCs versus aged-hADSCs, respectively). Additional marked increases were obtained when cells were treated with ML141 (~ 59- versus 62-fold, D−2/14 versus D14/28, respectively). The miR-122 inhibitor completely abolished the effects of ML141 on the expression of miR-122/ALB/HNF4α/E-cadherin, particularly when cells were treated with ML141(D−2/14); moderate effects of NSC5476 were seen in the case of ML141(D14/28) (Fig. 8a). Thus, for the following results, we will show only the data for ML141(D−2/14).

Second, the involvement of MAPK/PI3K and/or Wnt(s) in the regulation of miR-122 was evaluated (Fig. 8b). Cells were treated as previously described with: 1) the inhibitors of JNK/ERK/PKA/PI3K (SP600125, PD98059, H-89, Wortmannin (WRT), respectively); or 2) Wnt-antagonist (Dkk1) or Wnt5a, each for 24 h before adding ML141 and maintained throughout the ML141 incubation. SP600125, PD98059, H-89, and DKK1 did not show any blockade to the action of ML141 nor any additive effects, indicating no requirement for their pathways in this cell model to regulate miR-122 (Fig. 8b). However, Wnt5a significantly induced the expression of miR-122 in contrast to WRT which completely abolished the effect of ML141 on miR-122 expression (Fig. 8b). Interestingly, when cells were treated with Wnt5a + WRT, the PI3K inhibitor completely blocked the impact of Wnt5a on miR-122 expression indicating that PI3K acts as a target of Wnt5a in the regulation of miR-122 in the ML141-induced Hep-Dif of hADSCs (Fig. 8b)*.*

Third, the impact of miR-122 inhibition by NSC5476 was evaluated on the cell functionality (Fig. 8c). As expected, NSC5476 completely abolished the levels of ML141-induced secreted albumin. In addition, we were interested to evaluate the potential of these cells to release exosomes, taking into consideration the recently described role of the MSC-derived exosomes as a new therapeutic strategy for liver disease [59]. Notably, exosome release increased significantly during Hep-Dif ,particularly in the young group but not in the aged group, and ML141 induced marked increases in the exosome release of the aged-treated group reaching ~ 27-fold of the increase at day 28 of the differentiation (73.92 ± 1.44 × 10^7^ exosomes/μl vs 9.24 ± 3.65 × 10^7^ vs 249.48 ± 4.12 × 10^7^, young vs aged vs aged+ML141, respectively). However, when aged cells were pretreated with NSC5476, ML141 did not have any impact and could not produce exosome release during the differentiation; the release of exosomes was completely blocked (Fig. 8c). This release of exosomes was Wnt5a- and PI3K-dependent; in fact, when aged cells were treated with Wnt5a, a significant increase in the release of exosomes was observed, reaching ~ 36-fold at day 28 of the Hep-Dif, and more importantly this increase was abolished in the case of NSC pretreatment. On the other hand, WRT blocked the positive impact of ML141 on the exosome release (Fig. 8c).

### Suppression of Cdc42 by siRNA in aged hADSCs enhances Hep-Dif

**Fig. 9:**
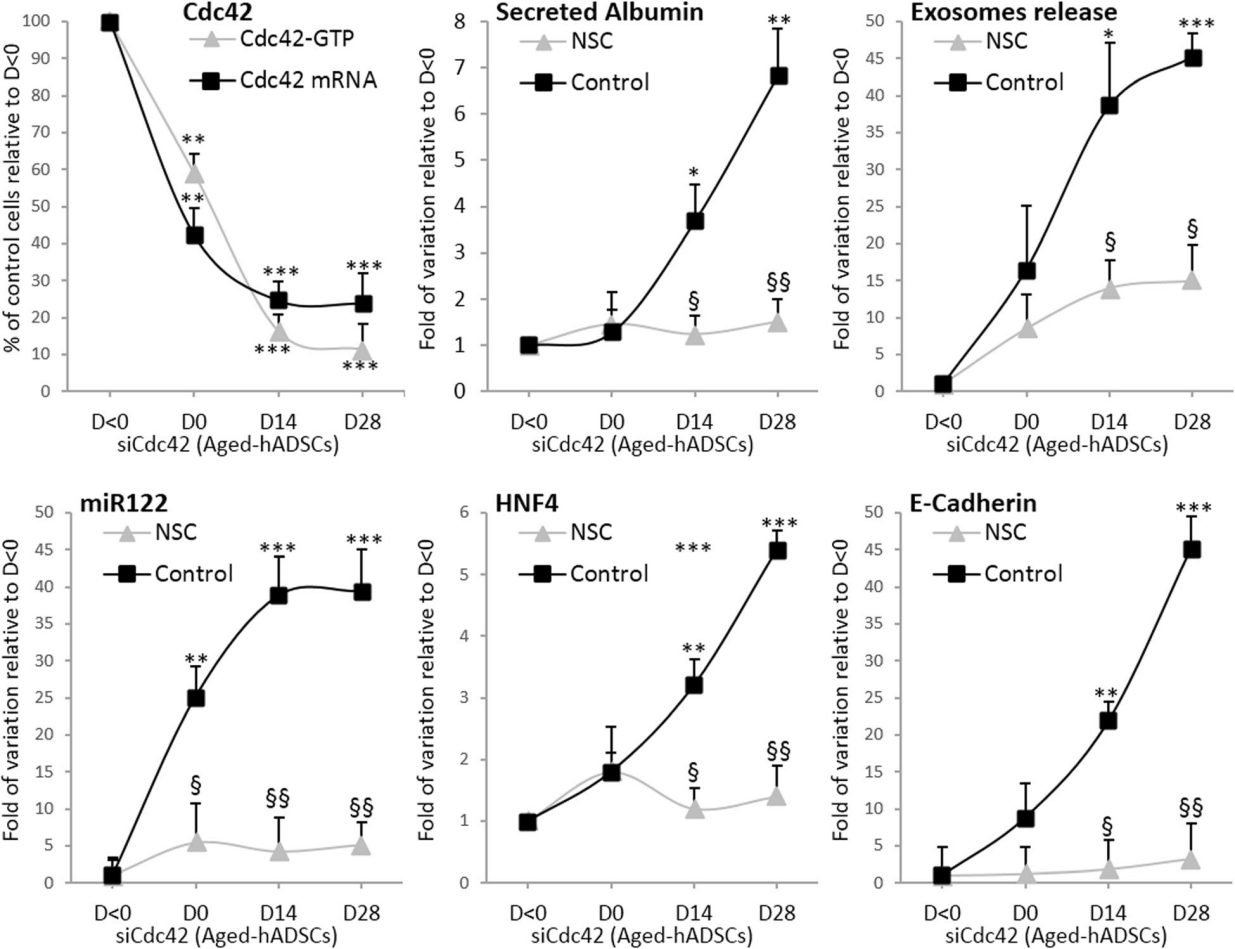
Induction of Hep-Dif in Cdc42 knockdown aged human adipose-derived mesenchymal stem cells (hADSCs). hADSCs derived from aged subjects were transfected with an Cdc42-targeting siRNA (siCdc42) at day –2 (D–2) and then differentiated at day 0 (D0) with 1% FBS. Cells were incubated with or without NSC5476 (NSC, 5 μM) 24 h before launching the differentiation and maintained along the Hep-Dif. Cdc42 mRNA and Cdc42-GTP protein levels as well as key factors were assessed during hepatocyte differentiation of Cdc42-knockdown cells from D<0 to D28: secreted albumin protein, released exosomes, miR122 mRNA, hepatocyte nuclear factor (HNF)4 mRNA, and E-cadherin mRNA. Results are normalized as previously to the expression of the housekeeping gene and are the mean ± SEM of three independent experiments and are expressed as the percentage or fold variation of control relative to day D–2 (considered as D<0). *P < 0.05, **P < 0.01, ***P < 0.005, D0/D14/D28 vs D < 0; ^§^*P* < 0.05, ^§§^*P* < 0.01, NSC vs control

Our data indicate that Cdc42 in its active form plays a key inhibitory role in Hep-Dif in hADSCs derived from elderly subjects, and this can be rescued by ML141. However, its direct role in hepatocyte differentiation of hADSCs needs to be confirmed. Thus, we investigated whether Cdc42 is essential for hepatogenesis of hADSCs. We transfected hADSCs derived from aged subjects with a Cdc42-targeting siRNA (siCdc42) in comparison with a negative control siRNA which has no targeting sequence. Several trials were performed to obtain a high effectiveness of the transfection assay, and the best result was obtained when undifferentiated cells were treated for 48 h with siRNA (D–2/0) and then were differentiated as previously but with 1% fetal bovine serum (FBS). Figure 9 shows that Cdc42 mRNA and Cdc42-GTP protein levels in aged cells transfected with siCdc42 were indeed significantly decreased. Concomitantly, siCdc42 significantly increased the secretion of albumin, the release of exosomes, and the mRNA expression of miR122/HNF4/E-cadherin. Conversely, by inhibiting miR122 with NSC5476, the effects of Cdc42 knockdown in aged cells were significantly abolished (Fig. 9) in favor of a mechanism of action involving the miR122 axis as shown with ML141.

### DNA methyltransferases regulate Hep-Dif of hADSCs

**Fig. 10:**
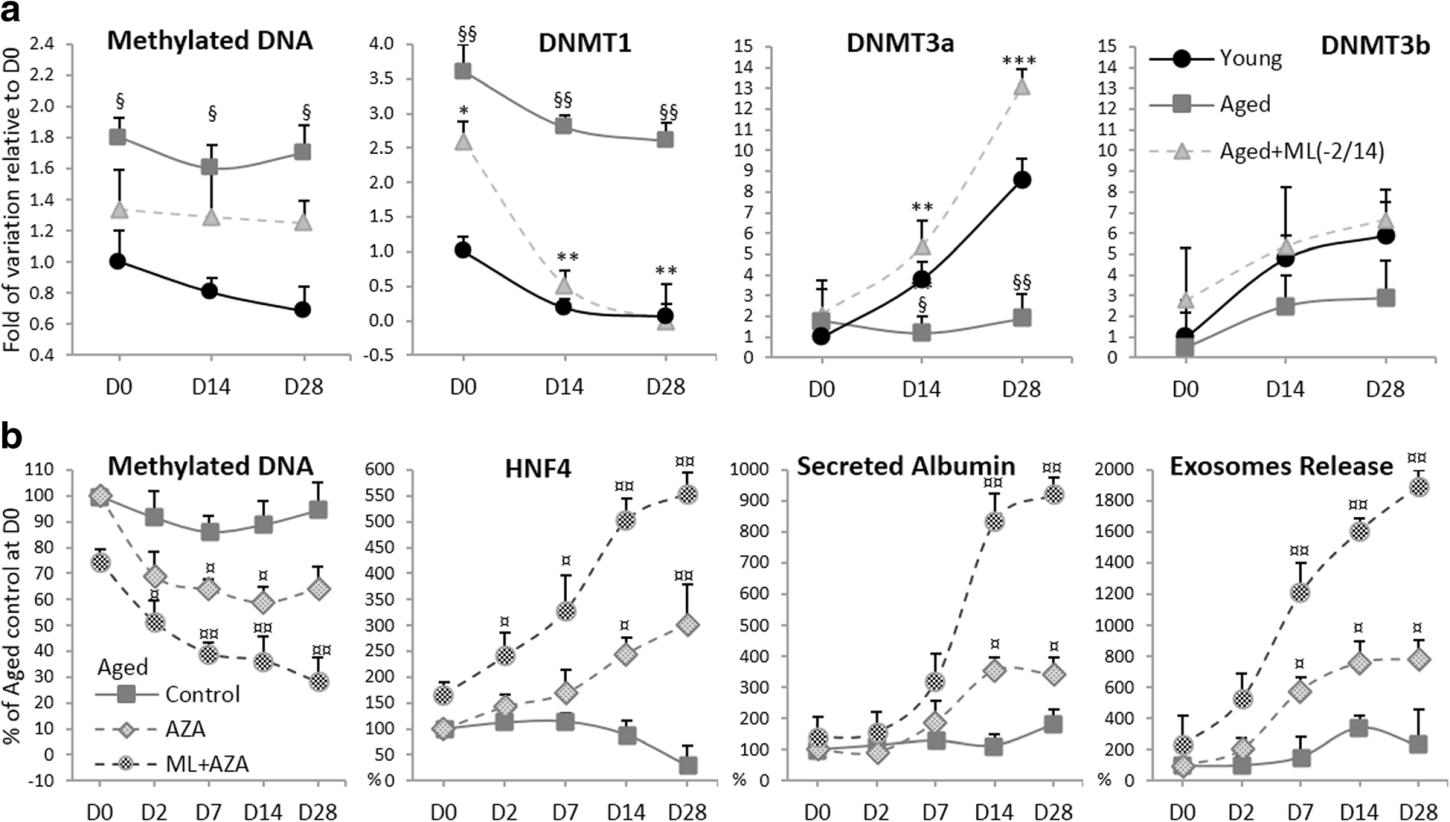
Global methylation status and involvement of DNA methyltransferases (DNMTs) in Hep-Dif of hADSCs. hADSCs derived from young and aged subjects were differentiated as indicated in Fig. 4. Cells were incubated with or without ML141 (ML, 10 μM) from day (D)−2 to D14 of the differentiation. **a** Methylation status of hADSCs derived from the young, aged, and ML141-treated aged groups were analyzed for the indicated time of Hep-Dif; expressions of DNMTs mRNA are shown. **b** hADSCs derived from aged subjects were subjected to Hep-Dif in the presence or absence of 5-azacytidine (AZA, 20 μM) for 48 h (from day 0 to day 2 of the differentiation)); ML141 was added from day −2 to day 14: methylation status, hepatocyte nuclear factor (HNF)4α mRNA, secreted albumin protein, and released exosomes are shown. Results are normalized as previously to the expression of the housekeeping gene and are the mean ± SEM of three independent experiments and are expressed as the percentage or fold variation of control relative to day D0. ^§^*^¤^*P* < 0.05, ^§§^**^¤¤^*P* < 0.01, ***P < 0.005; ^§^aged vs young, *aged+ML vs aged, and ^¤^AZA or AZA + ML vs control

Epigenetic changes, particularly DNA methylation, are considered to be one of the most important regulatory pathways affecting aging, stem cell aging, and MSC differentiation [60]. More interestingly, epigenetic changes support the hepatic differentiation and lead to an increase in metabolic and enzymatic activities [61]. Recently, it has been reported that DNA methyltransferases (DNMTs) modulate hepatogenic lineage plasticity of mesenchymal stromal cells [62]; DNMTs control gene transcription and cellular phenotypic changes during liver organogenesis [63], and inhibition of DNMTs increases liver-specific gene expression to maintain a hepatic fate in ADSCs [61]. Furthermore, a concomitant decrease in DNMT1 and increase in DNMT3 expression was associated with hepatic maturation [64]. Therefore, it was important to investigate whether the expression of DNMTs and DNA methylation are affected by ML141 treatment (Fig. 10a). Our results showed that the levels of global DNA methylation were significantly superior in the aged hADSCs and were maintained during their Hep-Dif in comparison with the young group, where a moderate demethylation was observed during Hep-Dif; however, no significant impact of ML141 was displayed. Interestingly, we found that DNMT1 expression decreased during Hep-Dif in the young-derived hADSCs to levels significantly lower than the Hep-Dif of the aged-derived hADSCs. In contrast, DNMT3a mRNA expression increased significantly during Hep-Dif in the young-derived hADSCs to levels significantly higher than the Hep-Dif of the aged-derived hADSCs. However, no significant variations were observed for DNMT3b, either in the young group or in the aged one. Importantly, only the ML141 treatment (D−2/14) significantly reversed the levels of expression of DNMT1 and DNMT3 in the aged-treated group.

Furthermore, it has been reported that the MSC immune-phenotype totally converts into the hepatic-phenotype upon differentiation by targeting DNA demethylation with 5-azacytidine (AZA), a pan-DNMT inhibitor [61, 62]. Pretreatment of hADSCs with AZA (20 μM, 48 h at D0) shortened hepatogenic differentiation times from 28 to 16–17 days in the young group but not in the aged group. The Hep-Dif time of aged hADSCs remained at 28 days but an increased efficacy of the differentiation was obtained. In fact, pretreatment of aged hADSCs with AZA reduced global DNA methylation during the 28 days of the Hep-Dif and improved the effect of ML141 (Fig. 10b). Over the differentiation period, AZA + ML141 enhanced hepatogenic-specific gene expression and hepatic functions with a higher effectiveness (Fig. 10b). These results indicated that DNMTs are involved in the Hep-Dif of hADSCs and the combined protocol of AZA + ML141 can provide better potential to recover the inhibited Hep-Dif in hADSCs derived from aged subjects, thus evidencing that the combination of epigenetic changes plus a distinct and specific ML141-induced differentiation protocol results in cells with specific hepatic features of aged-derived hADSCs.

## Discussion

**Fig. 11:**
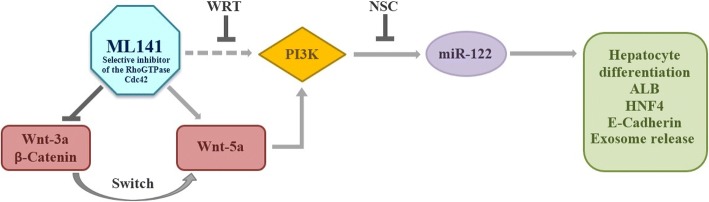
Model for the regulation of ML141-induced hepatocyte differentiation. In hADSCs, ML141 induced the hepatocyte differentiation by a mechanism involving the Wnt5a/PI3K/miR-122 signaling pathway, and regulated positively the hepatic specific genes and function, importantly the exosome release. ALB albumin, HNF hepatocyte nuclear factor, NSC NSC5476, PI3K phosphatidylinositol-3 kinase, WRT Wortmannin

In this study, our strategy was to screen for the first time the activity of Cdc42 in hADSCs isolated from healthy donors and to investigate the effects of selectively inhibiting its activity. We investigated the potential of selective pharmacological inhibition of Cdc42 by ML141 to reverse the aged phenotype of hADSCs into a younger phenotype, which may remove the blockade or inhibition of the Hep-Dif. The results of this study indicate that the inhibition of Cdc42 promotes the hepatic differentiation of hADSCs through a Wnt5a/PI3K/miR-122/HNF4α/albumin/E-cadherin-positive action (Fig. 11). In the presence of ML141, hepatocyte-like cells differentiated from hADSCs showed typical functional hepatic features, such as the expression of ALB, HNF4α, CK-18, TAT, G6P, and AFP (the early marker protein of hepatic differentiation), inducible cytochrome-dependent activity, cellular uptake of LDL, urea synthesis, and glycogen uptake. In addition, ML141 induced a mesenchymal-epithelial transition (MET) and epigenetic changes during Hep-Dif*.*

hADSCs have recently emerged as a promising tool for clinical application in regenerative medicine, where the age of the donor may strongly impact various hADSC properties, particularly cell expansion and differentiation [10, 11]. However, Kokai et al. [65] reported recently that ADSC function is maintained with age. In fact, although the cells isolated from elderly subjects did not completely lose their proliferative potential, nonetheless they showed lower rates of expansion in vitro, which makes them a weak tool for clinical autologous use. Even though the ADSC yield has been shown to decline in aged cells and they exhibit decreased migration and differentiation abilities with senescent properties [66, 67], our results indicate that this yield did not show a significant variability within the different age groups. However, significant declines in the proliferative, adherence, and differentiation potentials were shown. Our strategy of hADSC-induced Hep-Dif by ML141 provides additional evidence for the potential of hADSCs to generate functional hepatocyte-like cells (HLCs) and agrees with the potential for these cells in hepatic differentiation [7–9].

Several strategies have aimed to reverse the aging in human adult stem cells by inhibiting the Cdc42 activity. Cdc42 has been shown to be involved in cell proliferation, polarity, migration, and differentiation [21, 68], and its activity exhibited an increase in the GTP binding complex in HSCs derived from aged subjects [26]. In addition, Cdc42GAP deficiency promotes genomic instability and premature aging-like phenotypes [20]. A positive correlation was reported between the donor age and the ratio of Cdc42-GTP/total Cdc42 in HSCs and progenitor blood cells [19, 69]. Similarly, our results show that, in hADSCs, the activity of Cdc42-GTP increased with age which correlated positively with Cdc42-GTP and negatively with Cdc42GAP. On the other hand, the immunomodulatory ability of MSCs that are known to be linked with age have been reported to have some influences with Cdc42; in fact, proinflammatory cytokine profiles increased with aged MSCs, where TNF-α and IFN-γ secretion increased in BMSCs and umbilical cord-derived MSCs (UCMSCs) from aged patients and synergistically induced MSC deficiencies via several pathways, among them NFkB signaling [70]. Puls et al. [71] reported that exposure of fibroblasts to inflammatory cytokines such as TNF-α triggers the activation of Cdc42. Our findings show that the paracrine cytokine panel within aged-derived ADSCs is typically proinflammatory since TNF-α/IFN-γ/IL-17A/IL-12p70/IL-6/IL-2 levels are markedly increased and ML141 treatment succeeded the reversibility in favor of anti-inflammatory balance.

Interestingly, the use of the Cdc42 inhibitor ML141 on hADSCs has never been studied before. Other inhibitors have been studied in HSCs or ADSCs, such as CASIN and TSA. We realized a comparative study between ML141, CASIN, and TSA, where a significantly higher impact especially on undifferentiated hADSC properties was obtained with ML141 (data not shown). First, CASIN-treated aged HSCs exhibited a level of Cdc42-GTP similar to that found in HSCs from young donors [26]. To the contrary, CASIN impact was revealed to be strongly different in cancer stem cells by promoting their growth arrest and migration [72]. Second, TSA, the nonspecific inhibitor of Cdc42 activity and an HDAC inhibitor, was able to maintain the pluripotency expression in placenta-derived hMSCs, delay the appearance of aging signs [73], and promoted the proliferation and self-renewal of human UCMSCs [74]. In our study, ML141 promoted better population doubling and less toxicity, and a better proliferation rate and adhesion potential similar to the nontreated young ADSCs. Furthermore, ML141 was able to repress the expression of the apoptotic and senescence genes p16, p53, and p21 within the treated ADSCs, and promoted anti-inflammatory potential in the treated ADSC subpopulation by decreasing the TNF-α/IFN-γ/IL-17A/IL-12p70/IL-6/IL-2 secretion levels. ML141 was recently used as a potent Cdc42 inhibitor in cancer stem cells to downregulate epidermal growth factor receptor (EGFR) and redox/Fyn/c-Cbl signaling pathways [75, 76], and to regulate apoptosis in neuroblastoma cells [77]. Our results correlate closely with recent findings where ML141 was shown to inhibit Cdc42 activity not only in human BMSCs, but also in endothelial cells and cancer stem cells by downregulating several pathways implicated in aging and the polarity of stem cell processes [76–80]. Other studies reported that TSA may reverse the age impact within other human MSC subtypes and inhibits in-vitro differentiation of BMSCs into neuronal lineage cells [73, 74, 81]. Cdc42 inhibition was described to induce hepatic differentiation and maturation in UCMSCs involving CK-18 and other key hepatic genes [82, 83].

Since hADSCs have been shown to be a promising tool for hepatocyte generation in vitro, we conducted Hep-Dif protocols adapted from Yin et al. [84] where TSA (added from day 14 to day 28 of the differentiation) was used by Yin et al. and shown to be essential to promote in-vitro differentiation of hADSCs toward functional hepatocyte-like cells. Here, in parallel with ML141 treatment, we conducted TSA treatment as a control (added at the same concentration as Yin et al., but for two periods of incubation as for ML141 treatment (D–2/14 and D14/28)). The same strategy was performed as for ML141, studying all the parameters (genes, cell function, etc.). We observed a similar profile of results but, in comparison to TSA treatment, ML141 showed more efficacy for enhancing Hep-Dif of hADSCs (unpublished data). Our novel strategy consisted of replacing the TSA treatment by a specific and direct Cdc42-GTPase inhibitor, ML141, and to target not only the maturation step of the Hep-Dif, but also the early step and particularly the undifferentiated stage. Remarkably, our strategy was new and innovative in terms of i) following a two-step protocol of ML141 treatment within the Hep-Dif protocol and ii) evaluating an extensive range of cytokines, growth factors, hepatocytes maturation markers, and the MET process, and showing evidence of the mechanism controlling this differentiation, in addition to a method for rescuing the reduced potential of differentiating aged-derived ADSCs in comparison with the young group. Indeed, our results showed that young and ML141-treated aged ADSC groups showed a decrease in endodermic genes (Sox17), fetal liver genes (AFP, HNF6), and an increase in the hepatic master gene (HNF4α) and hepatic functional genes (ALB, Cyp3A7, TAT,G6P, CK-8, and CK-18). Conversely, at day 28, HLCs derived from nontreated aged ADSCs showed an incomplete fetal hepatic profile similar to the profile seen in TSA-treated HLCs generated by Yin and coworkers [84]. Our HLCs were functional and expressed high levels of albumin and Cyp450 upon rifampicin induction. They were also able to stock glycogen, to uptake LDL, and produce urea better than the HLCs derived from young donors, and they further efficiently reversed the levels of secreted cytokines. Our results also showed that ADSCs had a lower potential to release exosomes, where ML141 rescued this loss of functionality. This led us to consider the use of ML141 for aged ADSC treatment as a promising tool for new therapeutic strategies that use MSC exosomes in liver diseases [59]. MET was shown to be crucial when we transdifferentiate MSCs into hepatocytes; promotion of MET by inhibiting Rac1 accelerates the hepatic differentiation of MSCs [85] by a downregulation of Twist and Snail expression. Our results show that ML141 treatment of aged ADSCs gave rise to HLCs presenting a typical epithelial phenotype and high E/N-cadherin expression ratio. This MET correlates closely with the appearance of functional hepatic markers hardly expressed in nontreated aged ADSCs such as albumin, TAT, G6P, and CYP450. Moreover, the positive coexpression of CK-18 and E-cadherin was reported to be a crucial parameter for MET in murine hepatic and embryonic stem cell [86] and human induced pluripotent stem cell [87] differentiation into hepatocytes. Although selected histone-deacetylase inhibitors (such as TSA) could improve cell function in differentiated MSCs, they are not suitable for use because of their negative effects on cell proliferation due to DNA damage and cell-cycle inhibition [88].

Other recent studies have reported that MSC immune-phenotype can be totally converted into hepatic-phenotype upon epigenetic-targeted differentiation [61, 62]. In fact, epigenetic modifications of undifferentiated ADSCs by AZA, known as a DNMT inhibitor, resulted in cell cycle arrest, induction of apoptosis, and hepatic differentiation, leading to increases in metabolic and enzymatic activities of the differentiated hepatocytes [61, 62]. In our study, we first differentiated the hADSCs without inducing any changes at an epigenetic level; we found that methylated DNA decreased significantly with the Hep-Dif in the young-derived hADSCs concomitant with decreased DNMT1 but increased DNMT3a. The aged-derived cells showed the opposite regulation and reversibility was obtained by treating the cells with ML141(D−2/14). Our results confirm the role of DNMTs in the induction of hepatic differentiation in vitro. In fact, it has been reported that a decrease in DNMT1 and an increase in DNMT3 expression were associated with hepatic maturation [64] and DNMT1 knockdown shortened the hepatic differentiation time from 28 to 14 days [62]. Our data in hADSCs derived from young and elderly donors show that when cells were pretreated with AZA (48 h before D0) this shortened the hepatogenic differentiation time from 28 to ~ 17 days in the young group but not in the aged group (where DNMT1 and DNMT3a were increased and decreased, respectively). More importantly, ML141 treatment in combination with an AZA-pretreatment hepatocyte differentiation protocol showed higher effectiveness compared with ML141 alone by enhanced hepatogenic-specific gene expression and hepatic function and giving better potential to recover the inhibited Hep-Dif in hADSCs derived from aged subjects. On the other hand, the epigenetic modification of the genome is considered to be one of the most important regulatory pathways affecting stem cell aging. Yan et al. [89] reported that a decrease in DNA hydroxymethylation in ADSCs correlates with donor age and that treatment with AZA induced proliferation and improved the osteogenic differentiation potential in these cells, illustrating an approach that could be used to rejuvenate ADSCs from aged donors.

Our findings also describe a mechanism of action in ML141-induced Hep-Dif of hADSCs involving MAPK, PI3k, Wnt/β-catenin, and miR-122 pathways. Indeed, the PI3K/PKB, MAPK, and WNT signaling pathways were shown to be involved in Hep-Dif [29–31]. First, the MAPK pathway was shown to be implicated in MSC aging. Indeed, p38, JNK, and ERK pathways trigger the tumor suppressor gene p53 transcriptional program, ROS accumulation and apoptosis, and emphasize the role of ERK-dependent Cdc42 signaling [66, 90]. Also, PI3K can be triggered by IRS ligand binding to IGF, thus activating the JNK pathway and subsequently Cdc42-GTP binding. Moreover, insulin and IGFs enhance Hep-Dif from human embryonic stem cells via the PI3K/PKB pathway [28]. Binding of HGF to its receptor induces multiple biological responses by the downstream effectors PI3K, ERK1/2, and p38MAPK. In our study, inhibition of Cdc42 activity by ML141 induced p-ERK, p-JNK, and p-PKB, but not p38, during the Hep-Dif. In addition, ML141 induced the phosphorylation of the CREB, PPARγ, and FABP4, and inhibited Pref-1, known as key factors to induce Hep-Dif and Adp-Dif. In fact, CREB, CCAAT/enhancer-binding protein (C/EBPα), and other transcription factors were reported to be downregulated in aged undifferentiated hADSCs and displayed a sequential hepatogenic transdifferentiation role of ADSCs [91]. In our study, the second set of experiments using inhibitors of PKA, JNK, ERK, and PI3K revealed an important role only for PI3K/PKB signaling in the regulatory effects of ML141 on hepatic differentiation of hADSCs but not ERK/JNK/PKA. These results reveal the importance of all these pathways to promote Hep-Dif, and shows evidence for the first time that only the PI3K is required for the mechanism of action of ML141 in regulating Hep-Dif*.*

Second, the involvement of Wnt(s) signaling was reported in Hep-Dif where the inhibition of the Wnt/β-catenin signaling promoted Hep-Dif [35–38]. It seems that β-catenin may play a key role in the proliferation process. Inducing the translocation of β-catenin to the nucleus increased cell proliferation, and its stabilization alone leads to increased propensity toward cholangiocytes over hepatocytes [39]; otherwise the Wnt pathway is the major regulator of polarity and cell fate specifications [40–42]. Overexpression of HNF4α in hMSCs suppressed hepatocellular carcinoma development through downregulation of the Wnt/β-catenin signaling pathway [92].

Third, the mechanism of action of Cdc42 involved miR-122 in ML141-treated cells. By inhibiting miR-122 with the selective inhibitor NSC5476, blockade of the expression of HNF4α, albumin, and E-cadherin was seen, thus indicating that no more reversibility of the action of ML141 can then occur. In addition, by inhibiting miR-122, secreted albumin and exosome release were completely inhibited in the ML141-induced Hep-Dif*.* Our results reveal that miR-122 increased significantly during Hep-Dif, especially in the young group, and this is in accordance with previously reported studies on the positive implication of miR-122 in hepatic differentiation [24, 50]. miR-122 was reported to be a direct target of the LETFs-HNF4 which controls the Hep-Dif [48], and its overexpression promotes Hep-Dif through a miR-122/HNF4α-positive feedback loop [49, 50]. Interestingly, PI3K/PKB signaling has been demonstrated to positively regulate miR-122 [51]. Mechanistic studies using inhibitors of PI3K, PKB, and mammalian target of rapamycin (mTOR) in primary cultured rat hepatocytes resulted in significant suppression of the insulin-mediated elevation of miR-122 levels. In addition, by using a PI3K inhibitor in combination with Wnt5a, our data show that Wnt5a is required for the activation of PI3K, thus inducing miR122 in hADSC-derived Hep*-*Dif. The role of miR-122 in liver function and diseases has also been reported. miR-122 is considered a key factor and therapeutic target in liver disease [43–45] where loss of its expression has been associated with hepatocellular carcinoma [46] and its presence is essential as a host factor for hepatitis C virus replication [47].

The novelty of our study comes from the fact that currently there are no previous reports using ML141 to reverse the age-related aberrations in aged stem cells and to promote greater hepatogenic potential than young differentiated ADSC counterparts. These data suggest that inhibition of Cdc42-GTP activity might represent a novel target to rejuvenate not only HSCs but also aged ADSCs by altering their immunomodulatory effects, decreasing apoptosis, and improving the cell activity potential. Cdc42 activity can be pharmacologically targeted to rejuvenate aged ADSCs for MSC-based therapies and tissue engineering. Effectively, the use of Cdc42 knockdown hADSCs in our study confirms the impact of Cdc42 inhibition to promote hepatic differentiation of these cells. Clinical liver disease applications in the future will need new approaches for ex-vivo safe manipulation, including ADSC cultivation and direct hepatic fate within a short time. Thus, Cdc42 inhibition may also be important for converting the weak hepatogenic potential of aged ADSCs into functional and mature derived ADSC hepatocytes. In summary, we have provided in-vitro experimental data to show that ML141 induces hepatic differentiation. Additional proof of evidence of the impacts of ML141 in inducing hepatic differentiation and function in vivo is ongoing.

## Conclusion

The novel observation of this report is that reversibility of the impact of aging of human mesenchymal stem cells is possible with the use of the pharmacological inhibitor of the small RhoGTPase Cdc42, ML141. Treatment of hADSCs derived from aged donors with ML141 especially at early stages promotes greater hepatogenic potential than young differentiated ADSC counterparts. Differentiated HLCs have typical functional hepatic characteristics, such as expression of several hepatic markers, among them ALB/AFP/CK-18, CYP-dependent activity and inducibility, cellular uptake of LDL, urea synthesis and glycogen storage, and exosome release. The PI3K/Wnt5a/miR-122 signaling pathways play important major roles in the regulatory effects on hepatic differentiation of hADSCs and are involved in cell fate determination.

## Additional files

Additional file 1: **Table S1.** Primer sequences used for quantitative RT-PCR. (DOCX 19 kb)

Additional file 2: **Table S2.** Characteristics of the studied population (results are expressed as the mean ± SEM). (DOCX 21 kb)

## Abbreviations

AChE: Acetylcholinesterase
Adp-Dif: Adipocyte differentiation
ADSC: Adipose derived-mesenchymal stem cell
AFP: Alpha fetoprotein
ALB: Albumin
BMSC: Bone marrow-derived mesenchymal stem cell
C/EBP: CCAAT/enhancer-binding protein
CD: Cluster of differentiation
Cdc42: Cell division cycle 42
CK: Cytokeratin
CREB: cAMP response element-binding protein
CYP3A: Cytochrome P450 3A
DNMT: DNA methyltransferase
ECM: Extracellular matrix
EGFR: Epidermal growth factor receptor
ERK: Extracellular signal-regulated kinase
G6P: Glucose-6-phosphate
GAP: GTPase-activating protein
GAPDH: Glyceraldehyde 3-phosphate dehydrogenase
GM-CSF: Granulocyte/macrophage colony-stimulating factor
GTP: Guanosine triphosphate
hADSC: Human adipose-derived mesenchymal stem cell
HBLC: Hepatoblast-like cell
Hep-Dif: Hepatocyte differentiation
HGF: Hepatocyte growth factor
HLC: Hepatocyte-like cell
HNF: Hepatocyte nuclear factor
HSC: Hematopoietic stem cell
IALC: Immature adipocyte-like cell
IGF: Insulin-like growth factor
IL: Interleukin
IFN: Interferon
IRS: Insulin receptor substrate
JAK: Janus kinase
JNK: Jun N-terminal kinase
LDL: Low-density lipoprotein
MALC: Mature adipocyte-like cell
MAPK: Mitogen-activated protein kinase
MET: Mesenchymal-epithelial transition
miR-122: MicroRNA-122
MSC: Mesenchymal stem cell
Neu-Dif: Neural differentiation
NF-kB: Nuclear factor kappa beta
NLC: Neuron-like cell
NSC: NSC5476
NSP: Neurosphere
PAK: p21-activated kinase
PI3K: Phosphatidylinositol-3 kinase
PKA: Protein kinase A
PKB: Protein kinase B
PPAR: Peroxisome proliferator activated receptor
ROS: Reactive oxygen species
STAT: Signal transducer and activator of transcription
SVF: Stromal vascular fraction
TNF: Tumor necrosis factor
TSA: Trichostatin A
UCMSC: Umbilical cord-derived mesenchymal stem cell
Wnt: Wingless-type MMTV integration site family member
