# Supplementary material for: Inhibition of the RhoGTPase Cdc42 by ML141 enhances hepatocyte differentiation from human adipose-derived mesenchymal stem cells via the Wnt5a/PI3K/miR-122 pathway: impact of the age of the donor
Source: Stem Cell Res Ther. 2018 Jun 19;9:167. doi: 10.1186/s13287-018-0910-5 (PMC6009972; doi:10.1186/s13287-018-0910-5)
Supplement: Supplementary file 1 — Table S1. Primer sequences used for quantitative RT-PCR. (DOCX 19 kb) [file 13287_2018_910_MOESM1_ESM.docx]

**Table S1. Primer sequences used for quantitative RT–PCR**

| **Gene** | **Forward Primer 5’- 3’** | | **Reverse Primer 5’- 3’** |
| --- | --- | --- | --- |
| hAFP | | CTGCTGCAGCCAAAGTGAAG | CCAGCACATCTCCTCTGCAA |
| hALB | | AAGCAGATGTTCCCAAAGCG | GCAAAGCAGGCCTCCTTATC |
| hCK18 | | ATCTTGGTGATGCCTTGGAC | CCTGCTTCTGCTGGCTTAAT |
| hCK7 | | ATAAAAGGCGCGGAGTGTCC | TCCAGAAACCGCACCTTGTC |
| hCK8 | | TGTTCCCAGTGCTACCCTG | GCCGTGGTTGTGAAGAAGAT |
| hCXCR4 | | CTGGCCTTCATCAGTCTGGA | TCATCTGCCTCACTGACGTT |
| hcyp3A4 | | GAGCTGAGATTGCACCACTG | GGGTGTTGAGGATGGAATGC |
| hcyp3A7 | | GGCTATCACAGATCCCGACA | TTTCCGCTGGTGAATGTTGG |
| hFoxa1 | | AAGACTCCAGCCTCCTCAAC | CGTATGCCTTGAAGTCCAGC |
| hFoxa2 | | ATTGCTGGTCGTTTGTTGTGG | GTACATGGGGCTCATGGAGTT |
| hFoxa3 | | AGTGGAGCTACTACCCGGAG | ACCTTGACGAAGCAGTCGTT |
| hG6Pc | | ATTGACACCACACCCTTTGC | GACGTAGAAGACCAGCTCGA |
| hGATA 4 | | CGACACCCCAATCTCGATATGT | TTTGGATCCCCTCTTTCCGC |
| hHNF1 α | | CAGAGTGTGCCGGTCATCAA | GAGGTGAAGACCTGCTTGGT |
| hHNF1 β | | CATACTCTCACCAACGGCCA | ACTGTCTGGTTGAATTGTCGG |
| hHNF4 α | | GGTGTTGACGATGGGCAATG | CTCATTCTGGACGGCTTCCTA |
| hHNF6 | | GCTTAGCAGCATGCAAAAGGAA | ACACCTTCGTGGCATGGTAG |
| hSox17 | | TTCATGGTGTGGGCTAAGGA | CCGGTACTTGTAGTTGGGGT |
| hTAT | | GAGTCAGCGCATTTTGGGAC | TCGGGTACTCAAAGCACGTT |
| hp16INKa | | TGACTCCCTCCCCATTTTCC | TTTTGGAGAGTCGGACTGCT |
| hp53 | | TGGCCATCTACAAGCAGTCA | GGTACAGTCAGAGCCAACCT |
| hp21 | | CCCAAGCTCTACCTTCCCAC | CTGAGAGTCTCCAGGTCCAC |
| Wnt3a | | CCACACCGTCAGGTACTCCT | TGTAGCTGGATGGAGTGCAG |
| Wnt4 | | ACAGCTGGAAGGCTGACAGT | TGCATGTCCTTCTCACAAGC |
| Wnt5a | | ATACTGGCTGACCACCTTGG | GACAAAGGCCTCAGAAGCAC |
| Wnt7a | | CCCTGAAGCTTACTGCTTGG | GCTACGATGTATGGGGCACT |
| Wnt11 | | CACCCCCAGATAGTTGTGCT | GAGGAGGAAAGCGACACAAG |
| β-catenin | | GAAACGGCTTTCAGTTGAGC | CTGGCCATATCCACCAGAGT |
| DNMT1 | | GTGGGGGACTGTGTCTCTGT | TGAAAGCTGCATGTCCTCAC |
| DNMT3a | | CCGGAACATTGAGGACATCT | CAGCAGATGGTGCAGTAGGA |
| DNMT3b | | TTGAATATGAAGCCCCCAAG | GGTTCCAACAGCAATGGACT |
| hGADPH | | AGCTCATTTCCTGGTATGACAAC | GTGGTCCAGGGGTCTTACTC |
