# Supplementary material for: Inhibition of the RhoGTPase Cdc42 by ML141 enhances hepatocyte differentiation from human adipose-derived mesenchymal stem cells via the Wnt5a/PI3K/miR-122 pathway: impact of the age of the donor
Source: Stem Cell Res Ther. 2018 Jun 19;9:167. doi: 10.1186/s13287-018-0910-5 (PMC6009972; doi:10.1186/s13287-018-0910-5)
Supplement: Supplementary file 2 — Table S2. Characteristics of the studied population (results are expressed as the mean ± SEM). (DOCX 21 kb) [file 13287_2018_910_MOESM2_ESM.docx]

**Table S2.** Characteristics of the studied population (results are expressed as the mean ± SEM)

| **Healthy Group (N = 61)** | **YOUNG** | **MIDDLE** | | **AGED** | | |
| --- | --- | --- | --- | --- | --- | --- |
| Number of subjects | 19 | 22 | | 20 | | |
| Sex | Female 100% | Female 100% | | Female 100% | | |
| Mean Age (years) | 23.8 ± 0.4 | 40.8 ± 0.6 | | 57.6 ± 0.9 | | |
| Adipose tissue (mg) | 342.1 ± 78.4 | 299.4 ± 63.6 | | 390.0 ± 87.4 | | |
| Number of isolated SVF cells | 254.3 ± 23.7 x 10^6^ | 210.2 ± 18.5 x 10^6^ | | 288.6 ± 21.4 x 10^6^ | | |
| Number of MSCs / mg tissue | 743.5 ± 102.7 x 10^3^ | 702.1 ± 91.4 x 10^3^ | | 738.3 ± 45.3 x 10^3^ | | |
| Body mass index (kg/m^2^) | 30.2 ± 1.1 % | 33.0 ± 2.8 % | | 31.4 ± 3.7 % | | |
| Never smoking | 12.1 ± 0.9 % | 41.9 ± 3.3 % | | 18.2 ± 4.5 % | | |
| Ex-smoking | 68.4 ± 3.7 % | 47.2 ± 6.5 % | | 61.6 ± 7.1 % | | |
| Current smoker | 19.5 ± 4.5 % | 10.9 ± 4.2 % | | 20.3 ± 3.0 % | | |
| Physically active ^(1)^ | 42.0 ± 6.6 % | 49.8 ± 5.7 % | | 40.5 ± 8.6 % | | |
| Healthy subjects | 19 out 19 | 22 out of 22 | | 20 out of 20 | | |
|  |  | |  | |  |  |
| **Exclusion criteria** |  | | | | |  |
| Total Cholesterol (mg/dL) | > 250 | | | | |  |
| Triglycerides (mg/dL) | > 200 | | | | |  |
| LDL^(2)^ cholesterol (mg/dL) | > 150 | | | | |  |
| HDL cholesterol (mg/dL) | < 35 | | | | |  |
| Fasting glucose (mg/dL) | > 120 | | | | |  |
| Fasting plasma insulin (μU/mL) | > 10 | | | | |  |
| Systolic blood pressure | > 140 mm Hg | | | | |  |
| Diastolic blood pressure | > 90 mm Hg | | | | |  |
| Inflammatory tissue | Previously- or currently- untreated (treated < 6 months) | | | | |  |
| Liver, GIT, metabolic, neurological, cardiac, renal disorders, diabetes | Previously- or currently- treated or untreated | | | | |  |
| CAD or Stroke | Previously- or currently- treated or untreated | | | | |  |
| Body mass index (kg/m^2^) | < 28 and > 35 | | | | |  |

1. *Physically active: walking or doing other kinds of exercise at least once per week or self-report of moderate/intense level of activity in daily life.*
2. *Abbreviations: LDL: low density lipoprotein; HDL: high density lipoprotein; CAD: coronary artery disease; SVF: Stromal Vascular Fraction*
